# Lipidomic-based investigation into the regulatory effect of Schisandrin B on palmitic acid level in non-alcoholic steatotic livers

**DOI:** 10.1038/srep09114

**Published:** 2015-03-13

**Authors:** Hiu Yee Kwan, Xuyan Niu, Wenlin Dai, Tiejun Tong, Xiaojuan Chao, Tao Su, Chi Leung Chan, Kim Chung Lee, Xiuqiong Fu, Hua Yi, Hua Yu, Ting Li, Anfernee Kai Wing Tse, Wang Fun Fong, Si-Yuan Pan, Aiping Lu, Zhi-Ling Yu

**Affiliations:** 1 Centre for Cancer and Inflammation Research, School of Chinese Medicine, Hong Kong Baptist University, Kowloon Tong, Hong Kong, China; 2 Institute of Integrated Bioinfomedicine & Translational Science, HKBU Shenzhen Research Institute and Continuing Education, Shenzhen, China; 3 Institute of Basic Research in Clinical Medicine, China Academy of Chinese Medical Sciences, China; 4 Department of Mathematics, Hong Kong Baptist University, Kowloon Tong, Hong Kong, China; 5 Agilent Technology, Hong Kong Limited, Hong Kong, China; 6 Department of Pathology, Guangzhou University of Chinese Medicine, China; 7 Department of Pharmacology, Beijing University of Chinese Medicine, Beijing, China

## Abstract

Schisandrin B (SchB) is one of the most abundant bioactive dibenzocyclooctadiene derivatives found in the fruit of *Schisandra chinensis*. Here, we investigated the potential therapeutic effects of SchB on non-alcoholic fatty-liver disease (NAFLD). In lipidomic study, ingenuity pathway analysis highlighted palmitate biosynthesis metabolic pathway in the liver samples of SchB-treated high-fat-diet-fed mice. Further experiments showed that the SchB treatment reduced expression and activity of fatty acid synthase, expressions of hepatic mature sterol regulatory element binding protein-1 and tumor necrosis factor-α, and hepatic level of palmitic acid which is known to promote progression of steatosis to steatohepatitis. Furthermore, the treatment also reduced hepatic fibrosis, activated nuclear factor-erythroid-2-related factor-2 which is known to attenuate the progression of NASH-related fibrosis. Interestingly, in fasting mice, a single high-dose SchB induced transient lipolysis and increased the expressions of adipose triglyceride lipase and phospho-hormone sensitive lipase. The treatment also increased plasma cholesterol levels and 3-hydroxy-3-methylglutaryl-CoA reductase activity, reduced the hepatic low-density-lipoprotein receptor expression in these mice. Our data not only suggest SchB is a potential therapeutic agent for NAFLD, but also provided important information for a safe consumption of SchB because SchB overdosed under fasting condition will have adverse effects on lipid metabolism.

Nonalcoholic fatty liver disease (NAFLD) encompasses a wide spectrum of liver disorders, ranging from simple steatosis to steatohepatitis (NASH) which may present with increased hepatic fibrosis and leads to end-stage liver diseases such as cirrhosis, liver failure and hepatocellular carcinoma1^1^,^2^. The World Gastroenterology Organization reported that the worldwide prevalence of NAFLD was 20–34%, and the mortality of NASH patients reached 36%^3^. Metformin and thiazolidinediones (TZDs) are commonly used for NAFLD treatment^4^. However, the efficacy of metformin needs to be assessed^5^ and TZDs are often associated with side effects^4^,^6^.

Recent literature revealed that elevated free fatty acids (FFAs), especially saturated FFAs, play a pathogenic role in NAFLD^7^. The saturated FFAs not only serve as the precursor for triglycerides (TG) synthesis of but also trigger endoplasmic reticulum stress and lipoapoptosis in hepatocytes^8^,^9^. The saturated FFA-induced lipoapoptosis in hepatocytes depends on mitochondrial dysfunction, cytochrome c release and caspase activation^10^. Indeed, the caspase 3 activation and hepatocyte apoptosis are the prominent pathologic features of NAFLD and directly correlate with disease severity^11^.

Palmitic acid represents 80–90% of total fatty acids produced in a cell via *de novo* fatty acid synthesis^12^, in which, fatty acid synthase (FAS) and acetyl CoA carboxylase (ACC) are the critical enzymes. Both ACC-1 and FAS can be regulated at transcriptional level by sterol regulatory element-binding protein-1 (SREBP-1) under nutritional regulation. In NAFLD patients, expressions of hepatic ACC and FAS were upregulated^13^; and *de novo* fatty acid synthesis is generally increased^14^. More importantly, an increase in saturated-to-unsaturated fatty acid ratio in steatotic liver is usually considered as a risk factor for the progression to NASH^8^,^15^. Therefore, it is suggested that inhibitors of the *de novo* fatty acid synthesis pathway can serve as therapeutically significant agents to prevent the progression of hepatic steatosis to NASH.

Schisandrin B (SchB) is one of the most abundant and bioactive dibenzocyclooctadiene derivatives found in the fruit of *Schisandra chinensis*, which grows in many places such as the northern China, Japan, Eastern Russia, Korea and the Himalayas, and has been traditionally used as an adaptogen^16^. We have previously reported that SchB treatment reduced hepatic lipid contents in an acute mouse model of hypercholesterolaemia in which the hepatic triglyceride (TG) level was increased but the serum TG level was reduced^17^. Indeed, NAFLD is an obesity-related multifactorial disorder that always links to hypertriglyceridemia in patients^8^,^18^,^19^. In this study, to have a better understanding of the therapeutic effect of SchB on NAFLD, we used high-fat diet-fed mice which had both hepatic and plasma TG elevated which mimic the clinical situation. Furthermore, this study also delineated the mechanisms of action underlying the effects of SchB on lipid metabolism.

## Results

### SchB treatments affect hepatic and plasma TG levels in fasting and long-term HFD-fed mice

Previously, we found that SchB treatment reduced hepatic lipid contents in hypercholesterolaemic ICR mice^17^ but a single high dose of SchB increased hepatic and serum triglyceride levels in fasting mice^20^,^21^. Here, we first investigated the effects of SchB on lipid metabolism in different C57BL/6 mouse models described in the material and method section.

**Figure 1:**
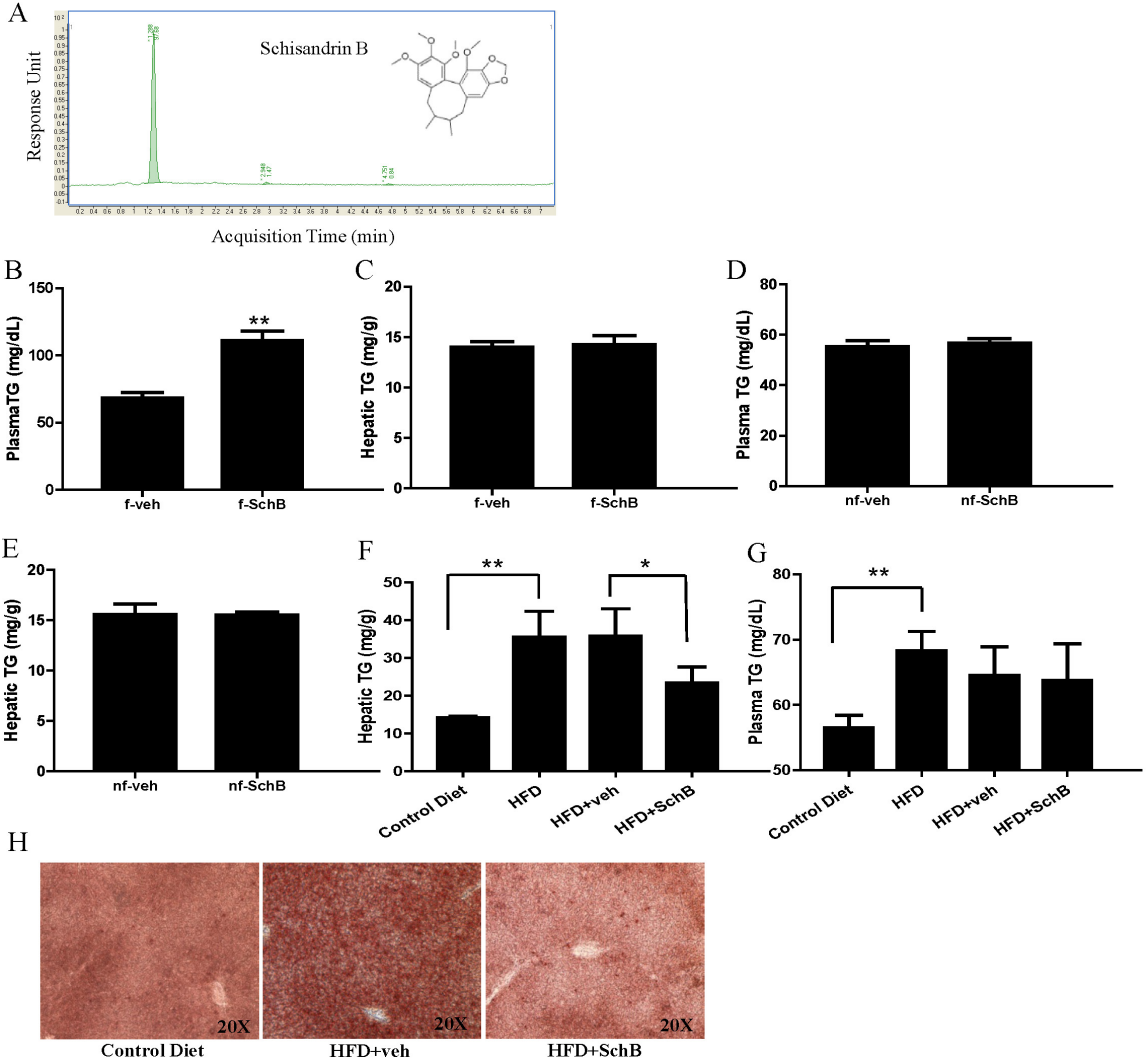
SchB treatments affect hepatic and plasma TG levels in mouse models. Schisandrin B (SchB) (A–B) the fruits of Schisandra chinensis; (C) SchB structure; (D) chromatogram of SchB in UHPLC analysis. (E) Plasma triglyceride (TG) and (F) hepatic TG in fasting group. (G) Plasma TG and (H) hepatic TG in non-fasting group. (I) Hepatic TG and (J) plasma TG in HFD-fed group. (K) Representative pictures show the Oil Red O staining of liver sections in HFD-fed mice. *nf-veh*: non-fasting vehicle control group; *nf-SchB*: non-fasting SchB-treated group; *f-veh*: fasting vehicle control group; *f-SchB*: fasting SchB-treated group; *HFD-veh*: HFD-fed vehicle control group; *HFD-SchB*: HFD-fed SchB-treated group. Shown is the mean ± SE (n = 10 mice in each group), *p < 0.05, **p < 0.01.

SchB is a dibenzocyclooctadiene derivative (Figure 1A). The SchB (Ningli Technology, China) used in this study was 97% pure as confirmed by UHPLC analysis (Figure 1A). SchB stock solution was dissolved in olive oil for *in vivo* experiments, and was in DMSO for *in vitro* experiments. Olive oil or DMSO alone was used as vehicle controls in these experiments, respectively.

In the fasting mice, we found that a single dose of SchB treatment at 0.8 g/kg significantly increased plasma TG level (Figure 1B), but not hepatic TG level (Figure 1C) when compared to its vehicle control mice. Interestingly, in the non-fasting mice, the same treatment did not have significant effect on both plasma (Figure 1D) and hepatic (Figure 1E) TG levels. Moreover, a single dose of SchB treatment at a lower dose (50 mg/kg) did not have any significant effects on plasma or hepatic TG levels in both fasting and non-fasting mice (data not shown). Nevertheless, we found that a 20-day administration of SchB at 50 mg/kg significantly reduced hepatic (Figure 1F) TG level, but not the plasma TG level (Figure 1G) in the HFD-fed mice when compared to the vehicle control mice. Oil Red O staining also showed that the SchB treatment significantly reduced hepatic neutral lipid contents with respect to its vehicle control (Figure 1H). The treatment did not have significant effect on liver weights (Supplementary Figure S1D) and SchB treatments did not significantly affect body weights (Supplementary Figure S1A to S1C) or food intake (data not shown) of the mice in these groups. Our results in this part of study suggest that SchB treatments affect plasma and hepatic TG levels in fasting and long-term HFD-fed mice (Table 1).

### Impacts of SchB treatments on lipidomic profiles in fasting and long-term HFD-fed mice

**Figure 2:**
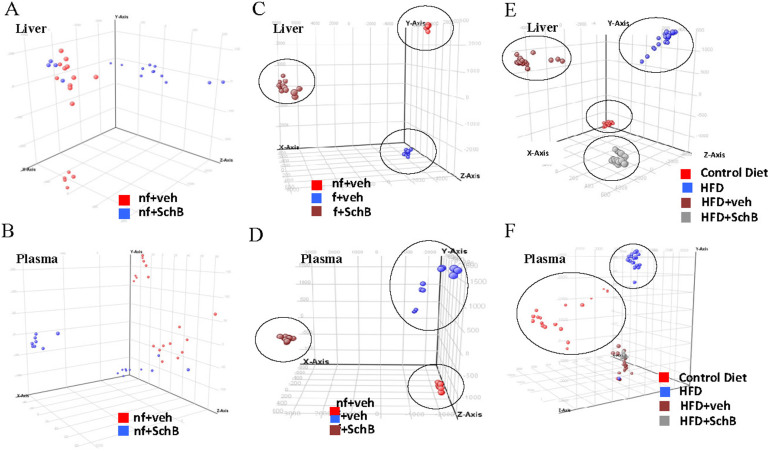
Impacts of SchB treatments on lipidomic profiles in mouse models. The Principle Component Analysis (PCA) of total lipids in (A) liver and (B) plasma samples in non-fasting mice, (C) liver and (D) plasma samples in fasting mice, (E) liver and (F) plasma samples in HFD-fed mice. *nf-veh*: non-fasting vehicle control group; *nf-SchB*: non-fasting SchB-treated group; *f-veh*: fasting vehicle control group; *f-SchB*: fasting SchB-treated group; *control diet*: control-diet-fed group; *HFD*: HFD-fed group; *HFD-veh*: HFD-fed vehicle control group; *HFD-SchB*: HFD-fed SchB-treated group.

Next, we employed LC/MS-based lipidomics analysis to explore the impacts of SchB on lipid metabolism in these mouse models. PCA demonstrated that non-fasting mouse liver (Figure 2A) and plasma (Figure 2B) samples did not show distinct clustering between vehicle and SchB treatment groups. Interestingly, fasting mouse liver (Figure 2C) and plasma (Figure 2D) samples showed distinct clustering between vehicle and SchB treatment groups, suggesting the SchB treatment or fasting affects the lipidomic profiles in these mice. The long-term HFD-fed mouse liver (Figure 2E) and plasma (Figure 2F) samples also showed distinct clustering between vehicle and SchB treatment groups, suggesting SchB treatment or the dietary intervention affects the lipidomic profiles in these mice. We have also identified the lipid entities in these samples that were differentially regulated by SchB treatments (Supplementary Table S2). In the fasting mice, among the 13 identified lipid species in the liver samples, 8 species were up-regulated, 5 were down-regulated; and among the 10 identified lipid species in the plasma samples, 7 species were up-regulated, 3 were down-regulated. In long-term HFD-fed mice, 7 species were down-regulated and 5 were up-regulated among the 12 identified species in the liver samples; and 1 was down-regulated with 4 up-regulated among the 5 identified species in the plasma samples.

### Metabolic pathway analysis with Ingenuity Pathway Analysis (IPA)

To further understand the physiological associations of these identified lipid species, we performed bioinformatics analysis using IPA software which led to the identification of the physiological association networks^22^. Supplementary Figures S2A and S2B show the built networks based on all the identified lipid species in liver and plasma samples in fasting mice, respectively. Supplementary Figures S2C and S2D show the built networks based on all the identified lipid species in liver and plasma samples in HFD-fed mice, respectively. To fully understand the impacts of SchB treatments on lipid metabolism in fasting and long-term HFD-fed mice, we also investigated the fatty acids and lipids metabolism canonical pathways identified by IPA in these mice for the liver and plasma (Table 2) samples, respectively. Interestingly, we found that palmitate biosynthesis metabolic pathway was highlighted in the liver samples of the fasting and HFD-fed mice but not in the non-fasting mice (Table 2). In the plasma samples, cholesterol biosynthesis and palmitate biosynthesis metabolic pathways were highlighted in the fasting mice but not in the HFD-fed and non-fasting mice (Table 2).

### SchB treatment reduces hepatic palmitic acid level in long-term HFD-fed mice

Since palmitate biosynthesis metabolic pathway is highlighted in the long-term HFD-fed mice in IPA, we next performed targeted lipidomics to examine the hepatic palmitic acid level in these mice. We found that SchB treatment significantly reduced hepatic palmitic acid levels by 12.26% in the HFD-fed mice; while the reductions in fasting mice (6.87%) and in non-fasting mice (2.61%) did not reach statistical significance (Table 3). These results suggest that hepatic palmitic acid level is significantly reduced in the long-term HFD-fed mice.

### SchB treatment reduces fatty acid synthase expression and activity in long-term HFD-fed mice

**Figure 3:**
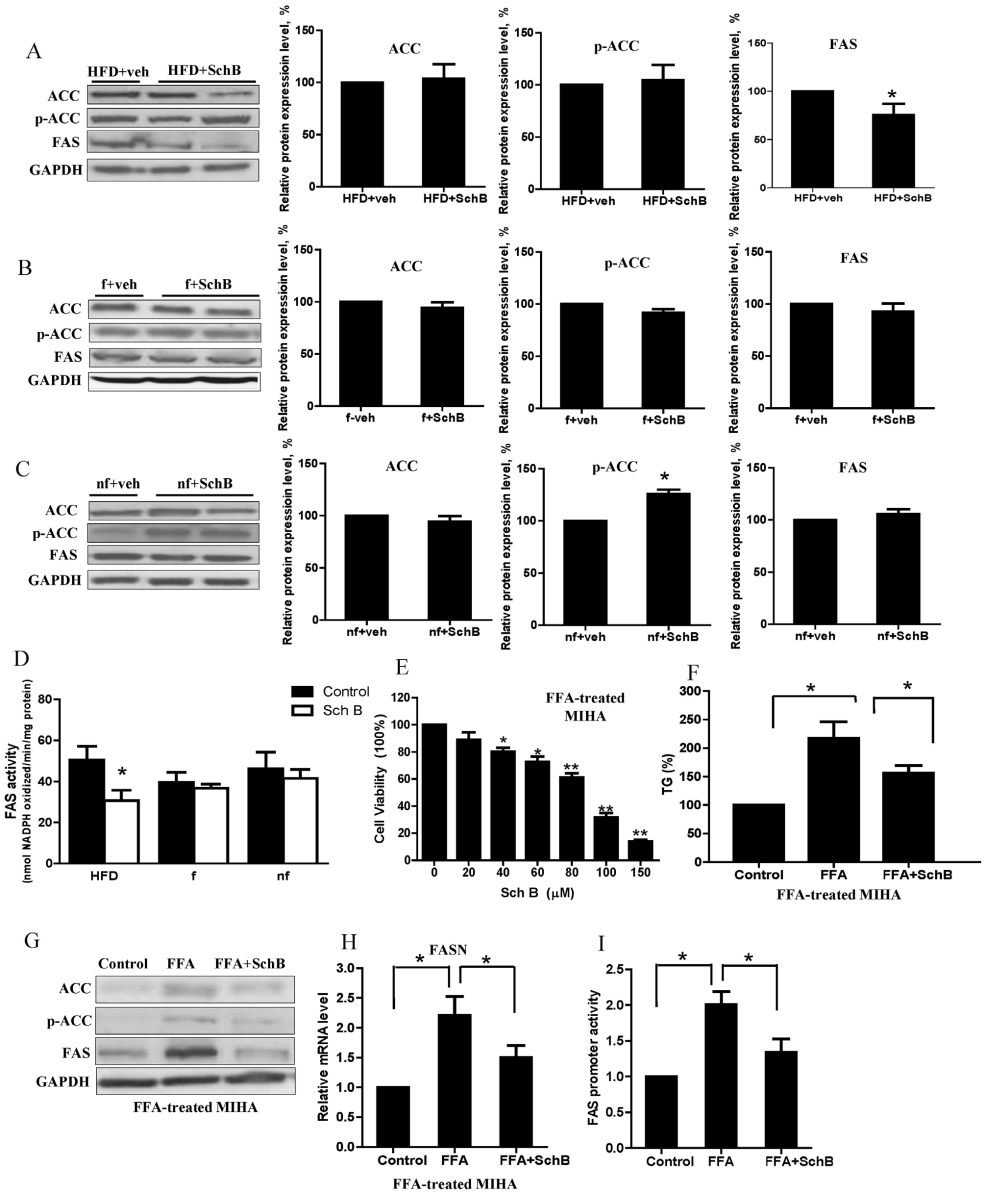
SchB treatment affects lipogenic gene expressions in long-term HFD-fed mice. Representative western blotting showing the expressions of acetyl CoA carboxylase (ACC), phospho-ACC (ser-563) and fatty acid synthase (FAS) in (A) HFD-fed mouse livers, (B) fasting mouse livers and (C) non-fasting mouse livers. Full length blots were shown in Supplementary S6. *nf-veh*: non-fasting vehicle control group; *nf-SchB*: non-fasting SchB-treated group; *f-veh*: fasting vehicle control group; *f-SchB*: fasting SchB-treated group; *HFD-veh*: HFD-fed vehicle control group; *HFD-SchB*: HFD-fed SchB-treated group. Bar charts showing the mean ± SE (n = 4 mice in each group), *p < 0.05. (D) Hepatic FAS activity of the mice. HFD: HFD-fed mice, f: fasting mice and nf: non-fasting mice. (E) MTT assay, (F) TG (% of control), (G) expressions of ACC, phospho-ACC (ser-563) and FAS. Full length blots were shown in Supplementary S6. (H) dual luciferase assay measuring the FAS promoter activity, and the (i) relative mRNA expressions of FASN in FFA-treated MIHA cells. *FFA*: FFA-treated vehicle control MIHA cells; *FFA+SchB*: FFA-treated SchB-treated MIHA cells. Shown is the mean ± SE (n = 3 independent experiments), *p < 0.05.

Next, we tried to find out the molecular target(s) of SchB in the palmitic acid biosynthesis pathway in the long-term HFD-fed mouse livers. Interestingly, we found that SchB treatments significantly reduced fatty acid synthase (FAS) protein expression (Figures 3A to 3C) and activity (Figure 3D) in the long-term HFD-fed mice, but not the fasting or non-fasting mice. In these HFD-fed mice, SchB treatment had no significant effects on total acetyl CoA carboxylase (ACC) or phospho-ACC expression levels (Figure 3A), suggesting FAS but not ACC is a molecular target of SchB in the palmitate biosynthesis in the long-term HFD-fed mouse livers. In the fasting mice, SchB treatment led to a reduction of 6.87% in the hepatic palmitic acid level (Table 3), we suggest that molecular target(s) other than FAS and ACC (Figure 3B) may be affected by the treatment. In the non-fasting mice, SchB treatment significantly increased the phosphorylation of ACC (Figure 3C), suggesting SchB treatment inhibits ACC activity. However, in the non-fasting mice, SchB treatment did not affect the FAS level (Figure 3C) and FAS activity (Figure 3D) and the hepatic palmitic acid level was only reduced by 2.6% (Table 3). We suggest that SchB treatment does not significantly affect the hepatic palmitic acid biosynthesis in the non-fasting mice. Indeed, the palmitate biosynthesis pathway was not highlighted in the non-fasting model in the IPA (Table 2).

We then used a cell model to further confirm the effects of SchB treatment on FAS expression in HFD-fed condition. We first induced lipid accumulation in MIHA cells by incubating the cells with FFAs mixture within the physiological range^23^ and then we used a sub-cytotoxic concentration of SchB (20 μM) (Figure 3E) to treat these cells. We found that SchB treatment significantly reduced FFA-induced TG accumulation (Figure 3F), FAS protein (Figure 3G) and mRNA (Figure 3H) expressions, and FAS promoter activity (Figure 3I). However, the treatment did not affect the mRNA expression levels of ACC, SCD-1 and elongation of long chain fatty acids family member 6 (ELOV6) (Supplementary Figures S3A to S3C). The reduction of TG accumulation upon SchB challenge is consistent with our previous study^24^.

### SchB treatment reduces expressions of hepatic mature SREBP-1 and tumor necrosis factor-α in long-term HFD-fed mice

**Figure 4:**
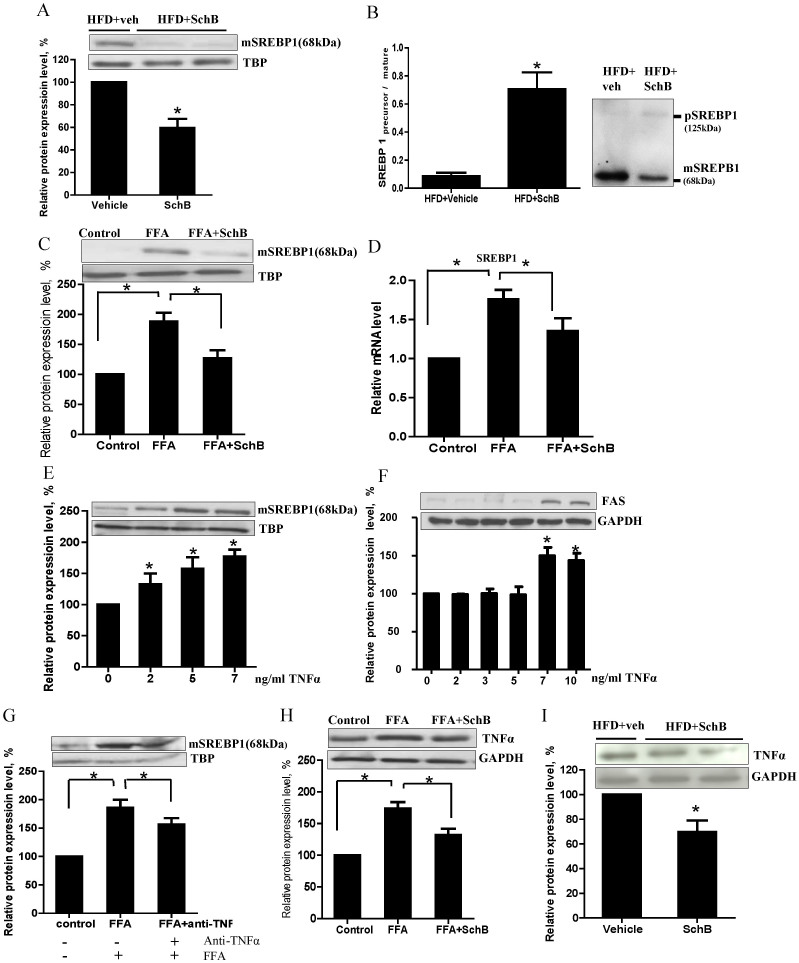
SchB treatment affects SREBP-1 expressions in long-term HFD-fed mice. Representative western blotting showing (A) expression of mature sterol regulatory element binding protein-1 (mSREBP-1), (B) ratio of precursor to mature SREBP-1 and (I) tumor necrosis factor (TNF-α) in HFD-fed mouse livers. Bar charts showing the mean ± SE (n = 4 mice in each group), *p < 0.05. (C) Protein expression of mature SREBP-1 (mSREBP-1) and (D) relative mRNA expression of SREBP-1 in FFA-treated MIHA cells. Protein expressions of (E, G) mSREBP-1 and (F) FAS upon TNF-α challenges, and (H) protein expressions of TNF-α in FFA-treated MIHA cells. Full length blots were shown in Supplementary S7. Bar charts showing the mean ± SE (n = 3 independent experiments), *p < 0.05. *FFA*: FFA-treated vehicle control MIHA cells; *FFA+SchB*: FFA-treated SchB-treated MIHA cells.

Interestingly, we also found that SchB treatment significantly reduced the hepatic nuclear mature SREBP-1 protein expression (Figure 4A) and increased the ratio of precursor SREBP-1 to mature SERBP-1 (Figure 4B) *in vivo* in the long-term HFD-fed mice.

In the FFA-treated MIHA cells, SchB treatment also reduced SREBP-1 protein (Figure 4C) and mRNA expression (Figure 4D). A previous study showed that SREBP-1 maturation was stimulated by tumor necrosis factor (TNF-α) in hepatocytes^25^. In our cell model, we also found that TNF-α challenge increased nuclear mature SREBP-1 protein expression (Figure 4E) and FAS expression (Figure 4F) in a dose-dependent manner, and the increased SREBP-1 expression was curtailed when these cells were pre-incubated with anti-TNFα (Figure 4G). These results suggest that TNF-α increase mature SREBP-1 and FAS expressions in the FFA-treated MIHA cells. Interestingly, we found that SchB treatment significantly reduced TNF-α protein expression in the FFA-treated MIHA cells (Figure 4H), and also in the livers of the long-term HFD-fed mice (Figure 4I).

Taken together, these data suggest that SchB treatment reduces SREBP-1 expression both *in vitro* and *in vivo*; the treatment also reduced the expressions of TNF-α, SREBP-1 and FAS.

### SchB treatment reduces hepatic fibrosis *in vivo* and activates factor-erythroid-2-related factor 2 (Nrf2)

**Figure 5:**
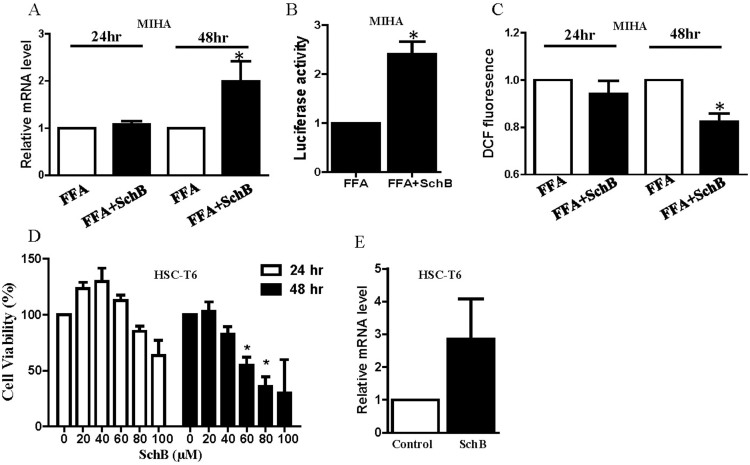
SchB treatment activates Nrf2 in FFA-treated cell model. (A) Expression of Nrf2 mRNA, (B) ARE-pGL6-luc reporter activity and (C) ROS levels in FFA-treated MIHA cells with or without SchB treatment (20 μM). (D) Cell viability examined by MTT assay and (E) mRNA expression level of Nrf2 in HSC-T6 cells with or without SchB treatment (40 μM). Bar charts showing the mean ± SE (n = 3 independent experiments), *p < 0.05. FFA: FFA-treated vehicle control cells; FFA+SchB: FFA-treated SchB-treated cells.

Indeed, nonalcoholic steatohepatitis (NASH) may present with increased hepatic fibrosis progressing to end-stage liver disease^1^. Recently, it is found that Nrf2 activation alone is sufficient to attenuate the progression of NASH-related fibrosis as demonstrated in Huh 7.5 cells and livers of NASH rat model^26^. Nrf2 is a well-known and essential transcription factor that regulates an array of detoxifying and antioxidant defense gene expression in liver^27^. Interestingly, we found that SchB treatment increased Nrf2 mRNA expression in FFA-treated MIHA cells (Figure 5A). Nrf2 binds to antioxidant response elements (ARE) within promoters of Nrf2-regulated genes and activates the gene transcription^28^. Here, we examined the transcriptional activity of Nrf2 after SchB treatment in FFA-treated MIHA cells that were co-transfected the ARE-luciferase reporter construct and Renilla luciferase (rLuc) expression vector (Promega). We found that SchB treatment significantly increased ARE-reporter luciferase activity as shown in Figure 5B. Activation of Nrf2 reduces reactive oxygen species (ROS) levels in liver^27^. Interestingly, SchB treatment significantly reduced ROS levels in the FFA-treated MIHA cells (Figure 5C). Furthermore, activation of hepatic stellate cell (HSC) also plays an important role in the pathogenesis of liver fibrosis in NASH^28^,^29^. Interestingly, SchB treatment significantly reduced rat HSC-T6 cell viability in both time- and dose-dependent manners (Figure 5D). The treatment also increased Nrf2 mRNA expression in HSC-T6 cells although the increase did not reach statistical significance (Figure 5E).

**Figure 6:**
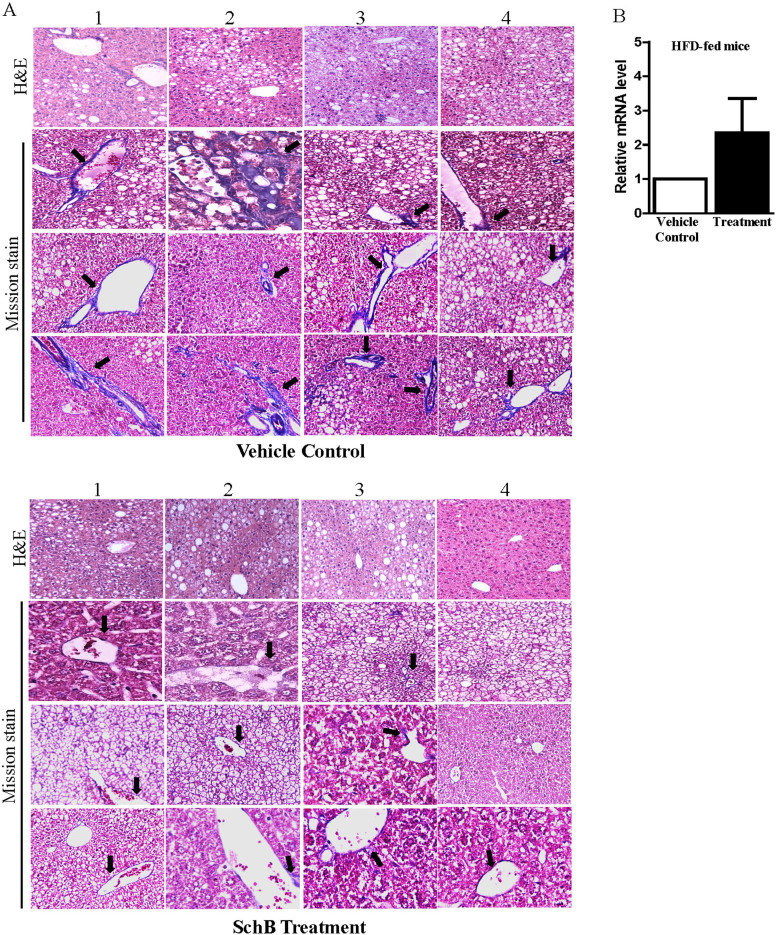
SchB treatment reduces hepatic fibrosis in HFD-fed mice. (A) Hematoxylin-eosin (H&E) stain and Masson's trichrome staining (Masson stain) in the liver samples of SchB-treated HFD-fed mice and vehicle control HFD-fed mice Three randomly selected Masson-stained microscopic fields on whole slide were presented (n = 4 mice in each group, labeled 1 to 4). Arrows indicated the Masson staining in color blue, original magnification ×100. (B) mRNA expression level of Nrf2 in the liver samples of SchB-treated HFD-fed mice and vehicle control HFD-fed mice (n = 4 mice in each group).

To further suggest the anti-hepatic fibrosis activity of SchB, we examined if SchB treatment reduced NASH-related hepatic fibrosis *in vivo*. Other studies showed that feeding mice HFD for 6 months led to NASH and hepatic fibrosis^30^,^31^. Here, after feeding mice HFD (Research Diets #D12492) for 6 months, we started intragastric administrating SchB at 50 mg/kg/day or vehicle as control for 14 consecutive days. After the treatment, we found that body weight and food intake between the SchB-treated mice and vehicle control mice did not have significant difference (data not shown). To investigate if SchB treatment reduced hepatic fibrosis, we stained the mouse steatotic liver samples by Masson's trichrome stain which stains type 1 collagen that is normally present in the vessel walls and portal tracts. Interestingly, the histological signs of hepatic fibrosis observed in the SchB-treated HFD-fed mice were obviously less when compared to those observed in vehicle control HFD-fed mice (Figure 6A). Furthermore, Nrf2 mRNA expression was higher in the liver samples of the SchB-treated HFD-fed mice when compared to those of the vehicle control HFD-fed mice (Figure 6B).

Taken together, our data suggest that SchB has a beneficial effect to the treatment of NASH-related fibrosis, at least in part, by activating the Nrf2-dependent pathways. However, further study is needed to fully elucidate the mechanisms underlying the effect of SchB in attenuating the progression of NASH-related fibrosis.

### SchB treatment increases plasma levels of fatty acids including palmitic acid in the fasting mice by inducing transient lipolysis

**Figure 7:**
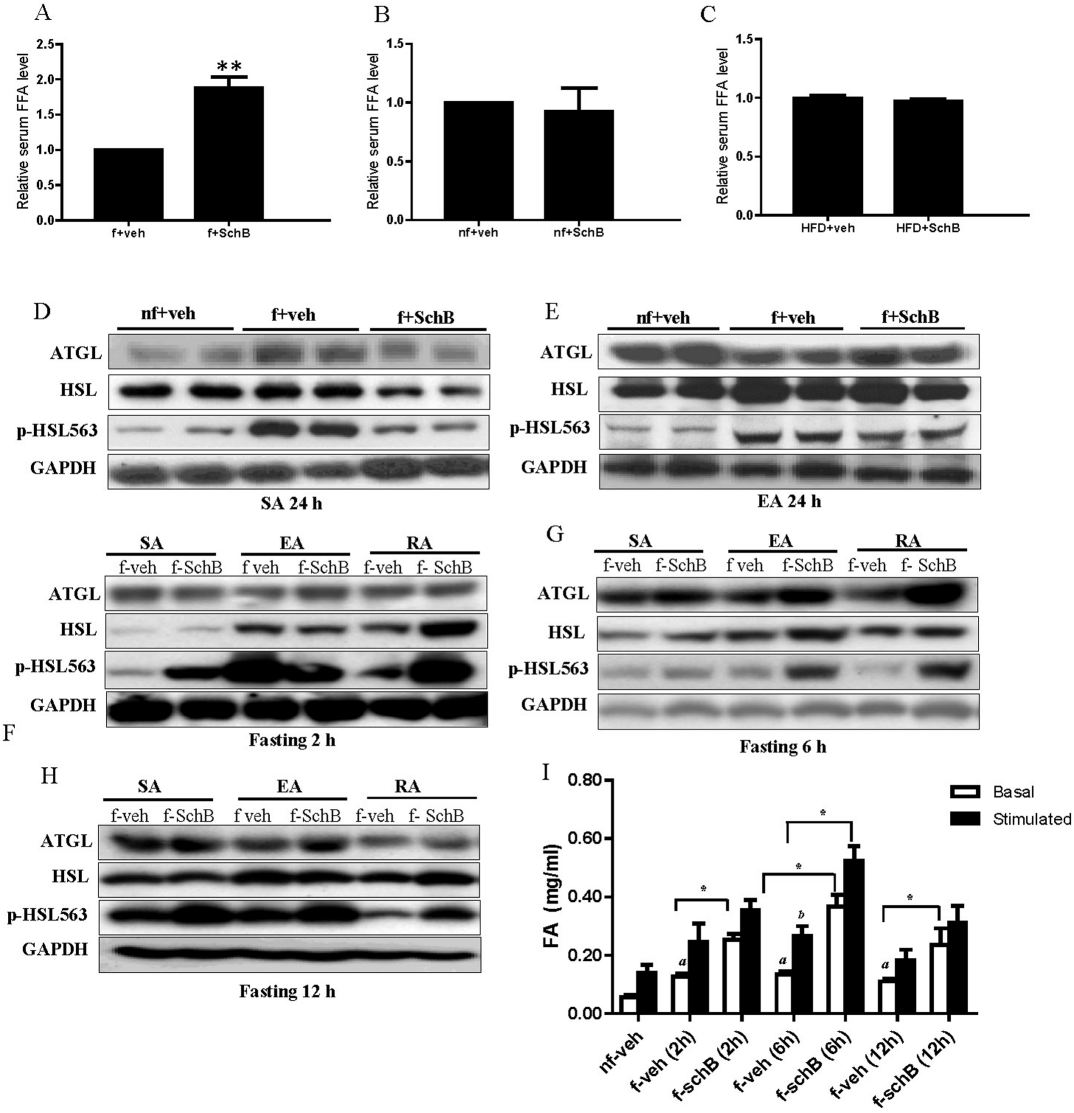
SchB treatment induces transient lipolysis in fasting mice. Relative free fatty acid (FFA) levels in (A) fasting mice, (B) non-fasting mice and (C) HFD-fed mice. Shown is the mean ± SE (n = 10 mice in each group), **p < 0.01. Representative western blotting showing the expressions of adipose triglyceride lipase (ATGL), hormone sensitive lipase (HSL), phospho-HSL (Ser-563) in adipocytes isolated from the bilateral superficial subcutaneous adipose tissue (SA), prominent bilateral intra-abdominal visceral depots attached to the epididymides (EA) and the perirenal fat (RA) (D–E) 24 h after fasting, (F) 2 h after fasting, (G) 6 h after fasting and (H) 12 h after fasting. Full length blots were shown in Supplementary S8. (I) The basal and isoproterenol (10 μM) stimulated fatty acids (FAs) released from subcutaneous adipose tissue dissected from fasting mice 2, 6 and 12 h after SchB treatment. Shown is the mean ± SE (n = 3 independent experiments), *p < 0.05. *a* < 0.05 compared to basal nf-veh. *b* < 0.05 compared to stimulated nf-veh. *nf-veh*: non-fasting vehicle control group; *nf-SchB*: non-fasting SchB-treated group; *f-veh*: fasting vehicle control group; *f-SchB*: fasting SchB-treated group; *HFD-veh*: HFD-fed vehicle control group; *HFD-SchB*: HFD-fed SchB-treated group.

IPA of the plasma samples highlighted the palmitate biosynthesis metabolic pathway in the fasting mice (Table 2). Therefore, we performed targeted lipidomics to investigate the plasma palmitic acid level in these mice. We found that SchB treatment significantly increased the plasma palmitic acid by 39.38% in these mice (Table 4). In the LC/MS-based lipidomic study, we also found that the total FFAs was significantly increased only in the fasting mice (Figures 7A–C).

The FFAs in plasma may come from adipocytes *via* lipolysis. However, in the fasting mice, we found that a 24 h-treatment of SchB reduced the expressions of ATGL, HSL and phospho-HSL in SA (Figure 7D) and reduced the expression of phospho-HSL in EA (Figure 7E). Interestingly, at 2, 6 or 12 hr after the SchB treatments, the expressions of phospho-HSL were significantly increased in SA, EA and RA (Figures 7F to 7H). ATGL expression was also increased 6 h after the SchB treatment in these adipocytes (Figure 7G). In parallel, we also found that both the basal lipolytic activities of the subcutaneous adipose tissue were significantly increased 2, 6 and 12 h after SchB treatments as measured by the FAs released from the tissues (Figure 7I); while the stimulated lipolytic activity was also significantly increased 6 h after the treatment (Figure 7I). These results suggest that SchB treatment induces a transient increase in lipolysis in the adipocytes in the fasting mice, which may partially explain the increase in the plasma FFA levels in these mice.

### SchB treatment affects the plasma cholesterol levels in fasting mice

**Figure 8:**
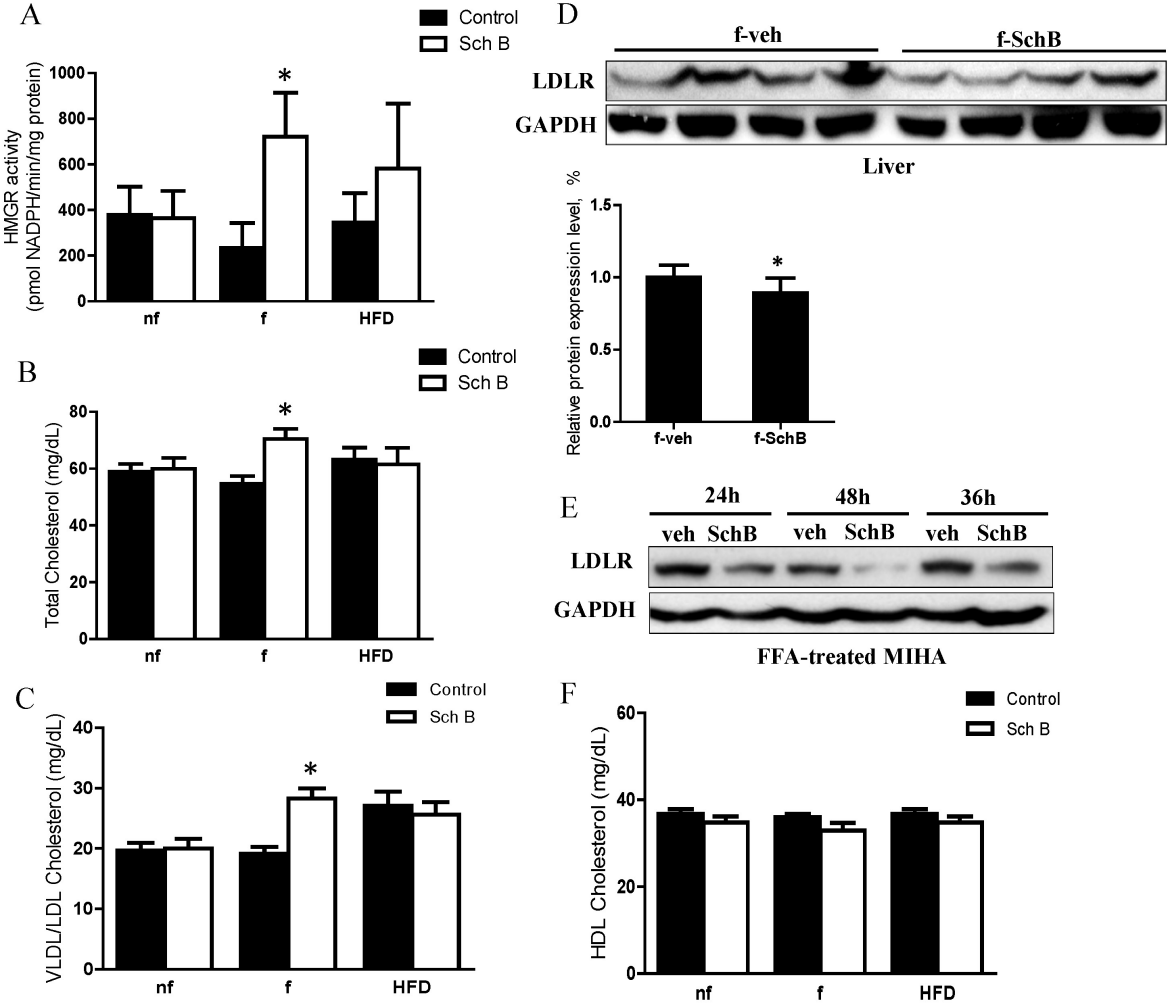
SchB treatment affects the plasma cholesterol levels in fasting mice. (A) 3-hydroxy-3-methylglutaryl (HMG)-CoA reductase activity, (B) total cholesterol, (C) VLDL/LDL cholesterol and (F) HDL cholesterol of non-fasting (nf), fasting (f) and HFD-fed (HFD) mice. Expressions of LDL receptor in (D) livers, bar charts showing the mean ± SE (n = 4 mice in each group), *p < 0.05, and in (E) FFA-treated MIHA cells, bar chart showing the mean ± SE (n = 3 independent experiments), *p < 0.05. *f-veh*: fasting vehicle control group; *f-SchB*: fasting SchB-treated group. Full length blots were shown in Supplementary S9.

Since IPA has highlighted the cholesterol biosynthesis metabolic pathway in the plasma samples of the fasting mice (Table 2), we tried to investigate the underlying mechanism. Interestingly, we found that SchB treatment significantly increased the HMG-CoA reductase activity (Figure 8A), the plasma total cholesterol level (Figure 8B) and VLDL/LDL cholesterol levels (Figure 8C). The treatment also significantly reduced hepatic LDL receptor expression (Figure 8D) in these fasting mice but not the non-fasting or HFD-fed mice (data not shown). In the FFA-treated MIHA cells, SchB treatment also reduced LDL receptor expression (Figure 8E). However, the SchB treatment did not have any significant effects on HDL cholesterol levels in the mouse models (Figure 8F). Taken together, we suggest that SchB treatment increases plasma cholesterol level in fasting mice, increases HMG-CoA reductase activity and VLDL/LDL cholesterol levels, and reduces hepatic LDL receptor expression.

## Discussion

**Figure 9:**
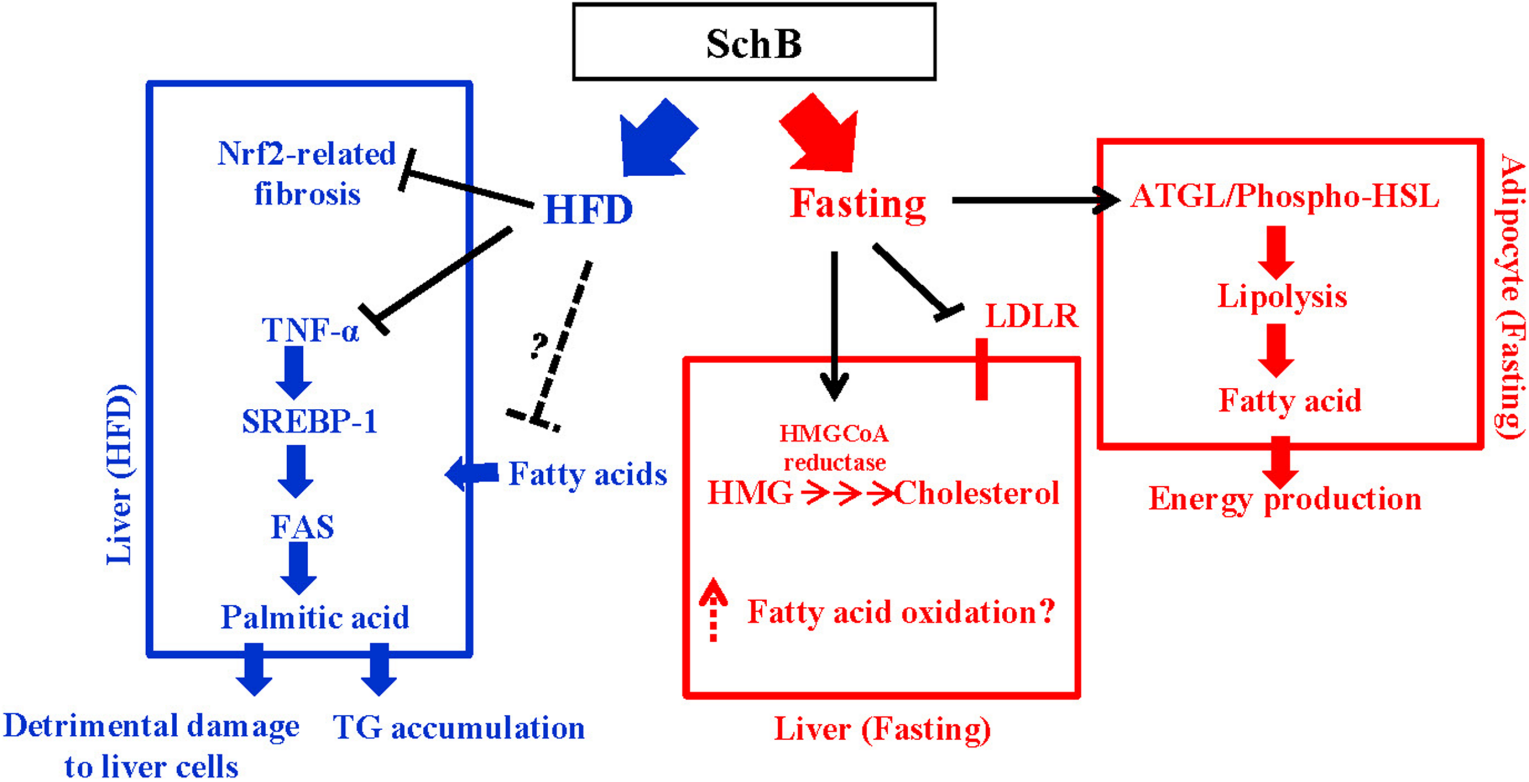
The regulatory roles of Schisandrin B on lipid metabolism under fasting condition and long-term high-fat diet feeding condition.

Here, we demonstrated that SchB treatment inhibited hepatic FAS expression and activity, reduced palmitic acid level in the steatotic livers under HFD-fed condition. The treatment also activated Nrf2 activity in FFA-treated cell model. However, under fasting condition, a single high dose of SchB treatment induced transient lipolysis, increased the levels of plasma FAs including palmitic acid; and also increased the plasma total and VDLD/LDL cholesterol levels, reduced LDL receptor expression and increased HMG-CoA reductase activity. The regulatory roles of SchB on lipid metabolism are summarized in Figure 9. Our data suggest that SchB may be a therapeutic agent to treat NAFLD. However, caution has to be taken because consumption of overdosed SchB under fasting condition will bring undesirable effects to the lipid metabolism.

To date, there is no broadly approved pharmacological therapy for NAFLD and no effective treatment for liver fibrosis is available^18^. Studies have examined the effects of insulin-sensitizing agents in patients with NASH. For example, pioglitazone, one of the most well established thiazolidinediones, is shown to improve liver histopathology in NASH. Antioxidant therapy with vitamin E also has a beneficial effect on the liver histopathology. However, the efficacies of these treatment are likely to be limited and have not yet been confirmed especially regarding fibrosis^18^,^32^,^33^. SchB is one of the most abundant and bioactive dibenzocyclooctadiene derivatives found in the fruit of *Schisandra chinensis*, which grows in many places such as the northern China, Japan, Eastern Russia, Korea and the Himalayas. Recently, it is reported that extract of fructus *Schisandrae chinensis* had protective role in hepatic injury in carbon tetrachloride-induced hepatic fibrosis rats^34^. Other studies showed that SchB had antioxidant activity^35^,^36^. Our studies suggest that SchB treatment reduces hepatic levels of triglyceride and palmitic acid which is known to induce lipotoxicity; the treatment also reduces hepatic fibrosis and activates Nrf2 pathway, which imply its beneficial effect to the treatment of NASH-related fibrosis. Furthermore, SchB is a natural compound, with relatively low toxicity^37^ and is also cheaper than synthetic compounds. All these suggest that SchB may be a potential new drug for NAFLD.

Interestingly, we found that the regulatory effects of SchB on lipogenic genes expressions and their enzymatic activities were different between high-fat diet-fed and fasting mouse models. It may be because the SchB treatment to HFD-fed mice lasted for a longer time compared to the treatments to fasting/non-fasting mice. Besides, one of the triggers for the differences in regulatory effects may be the difference in diet and/or nutrient availabilities for these mice. Indeed, lipogenic gene expressions and the subsequent lipid metabolism are affected by diet and/or nutrient availabilities. For examples, hepatic expressions of lipogenic genes and *de novo* lipogenesis was reduced during fasting^38^; however, in NAFLD patients, expressions of hepatic ACC and FAS were upregulated^39^ and *de novo* lipogenesis is generally increased^14^. Besides, saturated fatty acids in the HFD also increase lipogenic gene expressions^40^,^41^. Indeed, we found that HFD-fed mice had a higher expression of hepatic FAS (Supplementary Figure 4), which may, at least in part, explain why the inhibitory effect of SchB on FAS was more prominent in the HFD-fed mice. We also found that SchB treatment transient increased HSL phosphorylation in adipocytes isolated from fasting mice (Figure 5) but not HFD-fed mice (data not shown). Phosphorylation of HSL is mediated by stimulation of β-adrenoreceptor. In obese subjects, expression of β-adrenoreceptor in adipocytes is decreased and hence the phosphorylation and activation of HSL upon stimulation is partially impaired^42^, this phenomenon may help to explain why HSL phosphorylation in adipocytes isolated from HFD-fed mice was not increased after SchB treatment.

ACC catalyzes the irreversible carboxylation of acetyl-CoA to produce malonyl-CoA, and FAS subsequently condenses acetyl-CoA and malonyl-CoA to generate long chain fatty acids during lipogenesis. In the long-term HFD-fed mice, we found that SchB treatment reduced expressions of SREBP-1 and FAS, but not the expression of ACC in the livers. We suggest that the reduced expression of FAS is partially due to the reduced expression of SREBP-1. SREBP binds not only SRE but also E-box motif^43^ and SREBP activate the FAS promoter by binding to the -65 E-box during nutritional regulation^43^,^44^. Indeed, both the expressions of ACC and FAS are controlled by SREBP-1 but we could not detect a reduction of ACC expression in these liver samples. Others have studied the role of transcription in mediating diet-induced changes in the expression of ACC in rats. They found that, upon refeeding, the mRNA level of ACC increased by 9- to 12-fold while its transcription rate increased only by 2.5-fold; they suggested that the diet-induced changes in the abundance of ACC mRNA in the livers seem to be mediated primarily by a post-transcriptional mechanism^45^,^46^. Therefore, further experiments have to be done to examine the importance of SREBP-1 in upregulating ACC expression in our mouse models.

Indeed, SchB treatment may also mediate other molecular mechanisms to reduce hepatic TG in the HFD-fed mice. Dietary fat delivered by chylomicron remnants and plasma free fatty acids may also contribute to the liver TG^47^. Further investigation is needed to suggest other possible mechanisms that account for the reduction of hepatic TG in the SchB-treated HFD-fed mice.

Interestingly, we detected a significant decrease in hepatic TG but not the plasma TG in the HFD-fed mice. It has been reported that contribution of *de novo* lipogenesis to circulating VLDL triglyceride is undetectable or minor in subject fed with HFD^47^. Therefore, we suggest that the inhibitory effect of SchB on FAS unlikely causes a reduction of the plasma TG in the HFD-fed mice.

In this study, non-fasting mice received the same SchB treatment as the fasting mice. However, the SchB treatment did not have apparent effect on the lipid metabolism in these non-fasting mice. Previously, we reported that SchB treatment dose-dependently increased serum and hepatic TG levels in non-fasting ICR mice^48^. In this study, we used another strain of mice, the C57BL/6 mice. Reports suggest that different species^41^, or the same species of different strains^49^ may have different lipid metabolisms in response to the same treatment. For example, C57BL/6 mice were more sensitive to fasting as they showed a more significant reduction in lipogenic gene expressions and hepatic TG accumulation when compared to BALB/c mice^49^. Therefore, our previous^48^ and current data suggest that different strain of mice may have different lipid metabolism in response to SchB treatment under non-fasting regular diet-fed condition, which awaits further investigation.

Under fasting condition, SchB treatment reduced hepatic palmitic acid level by 6.8% (Table 3) although the decrease did not reach statistical significance. Study showed that expressions of genes involved in fatty acid oxidation in liver were increased after a 24-hour fasting^50^. Interestingly, we found that SchB treatment increased hepatic mRNA expressions of carnitine palmitoyltransferase-1 (CPT-1) and very-long-chain acyl CoA dehydrogenase (LCAH) (Supplementary Figure 5), implying fatty acid oxidation is increased in these livers after SchB treatment. If fatty acid oxidation in these livers is increased, the levels of hepatic fatty acids including palmitic acid may be reduced because the fatty acid will be used for energy production. Another study found that the association of [^14^C]-palmitate with liver mitochondria was enhanced from 190 to 330% in mitochondria isolated from fasted animals as compared to control animals^51^. Further study is needed to suggest if SchB treatment increases fatty acid oxidation in the livers in these fasting mice.

Here, we also found that SchB treatment activated Nrf-2 which is known to attenuate the progression of NASH-related fibrosis. Recently, it is found that Nrf2 activation alone is sufficient to attenuate the progression of NASH-related fibrosis as demonstrated in Huh 7.5 cells and livers of NASH rat model^27^. Indeed, other studies also demonstrated that SchB induced Nrf2 activation in lymphocytes^52^, and in hepatocytes under hypoxic condition^53^. SchB has been shown to reduce vascular fibrosis by suppressing transforming growth factor beta-1 (TGF-β1) signaling in vascular smooth cells^54^ and SchB is known to have antioxidant activity^35^,^36^.

Our data provide scientific evidence to suggest that SchB is a therapeutic agent to NAFLD treatment. Further experiment might suggest if SchB prevents the progression of hepatic steatosis to NASH and the NASH-related fibrosis. More importantly, the data also provided important information for a safe therapeutic use of SchB because SchB overdosed under fasting condition will have adverse effects on the lipid metabolism. This study sheds light on the future development of SchB as a therapeutic agent for the treatment of NAFLD.

## Methods

### Mice

All animal experimentation was conducted in accordance with the guidelines from Hong Kong Baptist University for the ethical use of animals, and was approved by the Hong Kong Baptist University Human and Animal Subject Committee and the Department of Health, the Government of Hong Kong Special Administration Region. Male C57BL/6 mice with body weights of 20–21 g were randomly divided into groups. Mice in the non-fasting group were fed regular diet (LabDiets #5001) with intragastric administration of single dose of SchB at 0.8 g/kg or olive oil as vehicle control. Mice in the fasting group were fed regular diet (LabDiets #5001) and were fasted for 24 h before intragastrically administrated a single dose of SchB at 0.8 g/kg or olive oil as vehicle control. Mice in the HFD group were fed either HFD (Research Diets #D12492) or control diet (Research Diets #D12450J), in parallel with daily intragastric administration of SchB at 50 mg/kg or vehicle as control for 20 consecutive days. After the treatments, animals were sacrificed. Blood was collected from the orbital vein, adipose and liver tissue samples were dissected for subsequent experiments. For the study of hepatic fibrosis, we used C57/BL mice that had been feeding HFD (Research Diets #D12492) for 6 months as a model^30^,^31^ to investigate if SchB treatment reduced NASH-related hepatic fibrosis. After we fed mice the HFD for 6 months, we started intragastric administrating SchB at 50 mg/kg or vehicle as control for 14 consecutive days. During the SchB treatment period, mice had free access to HFD and water.

### LC/MS analysis and statistical analysis

Lipids in the livers or plasma were extracted by Folch's method for LC/MS analysis^20^. The chromatographic and mass spectrometric parameters were shown in Supplementary Table 1. LC/MS raw data were processed by MassHunter Workstation software (version B.04.00 Qualitative Analysis, Agilent Technologies) and Mass Profiler Professional software package (version 2.2, Agilent Technologies) as described^20^. Authentic commercially available standards (Sigma-Aldrich), as shown in supplementary Table 2, were used to confirm the identities of the lipid species. Hexadecanoic-15,15,16,16,16-d5 acid was used as internal standard in the targeted lipidomics^20^.

### Ingenuity pathway analysis (IPA)

IPA was performed based on database sources including KEGG (http://www.genome.jp/kegg) and METLIN (https://metlin.scripps.edu/index.php) to identify the affected metabolic pathways^22^.

### Biochemical assays

Lipids were extracted by Folch's method and triglyceride was measured by commercial kit (Thermo Scientific). High-density lipoprotein (HDL) and very-low-density lipoprotein (VLDL)/low-density lipoprotein (LDL) cholesterol assays was performed by HDL and LDL/VLDL cholesterol assay kits (abcam) following company's instruction. All samples were analyzed in triplicate in each individual experiment.

### Cryosectioning

Fresh livers specimen was cut into slices at 8 μM and fixed before Oil Red O staining^55^.

### Masson's trichrome staining

Liver sections from paraffin-embedded tissues were prepared at 5-μm thickness. Liver pathology was examined by standard hematoxylin-eosin (H&E) staining (Sigma-Aldrich). Hepatic fibrosis was assessed using Masson's trichrome staining (Sigma-Aldrich) following company's protocol. All stained slides were examined by pathologist. Three randomly selected Masson's stained microscopic fields on whole slide were presented.

### FAS enzymatic activity assay

The FAS activity was measured as described^56^. Briefly, liver tissues were homogenized and centrifuged. Supernatant was added to 100 mM phosphate buffer (pH 6.5) containing acetyl-CoA and NADPH. The reaction was started by adding malonyl-CoA. Oxidation of NADPH was followed at 340 nm at 37°C for 20 min. All samples were analyzed in triplicate in each individual experiment.

### HMG-CoA reductase activity assay

The HMG-CoA reductase activity was measured as described^57^. Briefly, liver tissues were homogenized and centrifuged. The pellet was re-suspended in 0.1 M Tris buffer (pH 7.4) and was added to the reaction buffer containing 50 mM potassium phosphate, 0.5 mM MgCl_2_, 1.25 mM NAD^+^, 1.25 mM cysteine hydrochloride and 1 mM 2-oxoglutarate. The increase in fluorescence caused by NADH formation was recorded at 340 nm at 25°C for 20 min. All samples were analyzed in triplicate in each individual experiment.

### Isolation of adipocytes

Adipocytes were isolated from adipose tissues including the bilateral superficial subcutaneous adipose depots (SA), prominent bilateral intra-abdominal visceral depots in male mice attached to the epididymides (EA) and the perirenal fat (RA)^58^. Briefly, adipose tissues were digested for with collagenase in Krebs-Ringer Buffer (KRB) (12 mM HEPES, 121 mM NaCl, 4.9 mM KCl, 1.2 mM MgSO_4_ and 0.33 mM CaCl_2_) supplemented with 3 mM glucose and 1% fatty acid free BSA, and were filtered through nylon mesh^59^. Adipocytes were collected from the upper phase after centrifugation.

### Lipolysis assay

Adipose tissues were cut into 50 mg samples and incubated at 37°C without shaking in KRB containing 2% fatty acid free BSA and 0.1% glucose^59^ in the presence or absence of SchB or vehicle. At the indicated time point, NEFAs were measured in aliquots from incubation buffer using LabAssay NEFA kit (Wako Chemicals)^59^. All samples were analyzed in triplicate in each individual experiment.

### Cell culture

MIHA cells were incubated with or without 1 mM free fatty acids (FFAs) (2:1 oleate/palmitate, Sigma-Aldrich) in medium containing 1% free fatty acid-free BSA^24^ for 24 h before SchB or vehicle treatment. The FFAs used was within physiological concentrations^23^. HSC-T6 cell (ATCC, USA) is an immortalized activated rat hepatic stellate cell. HSC-T6 cells were cultured in DMEM medium supplemented with 10% fetal bovine serum.

### Cell viability assay

Cytotoxicities of SchB to MIHA cell and HSC-T6 cells were assessed by 3-(4,5-dimethylthiazol-2-yl)-2,5-diphenyltetrazolium bromide assay. All samples were analyzed in triplicate in each individual experiment.

### Real-time polymerase chain reaction

Total RNA was isolated from MIHA cells and treated with DNAase1 (Invitrogen). Complementary DNA was prepared using SuperScript VILO cDNA synthesis kit (Invitrogen). Real-time PCR was performed using SYBR green reaction mixture in the ABI 7500 fast real-time PCR system (Applied Biosystems). All samples were analyzed in triplicate in each individual experiment.

### Western blotting

Immunodetection was performed by ECL detection system (Amersham). Nuclear protein was extracted by NE-PER nuclear and cytoplasmic extraction kit (Thermo Scientific) following company's instruction.

### Transient reporter assay

The human FAS promoter reporter construct (FAS-luc) was kindly given by Prof Qiang Liu (University of Saskatchewan, Canada)^60^. MIHA cells were co-transfected FAS-luc and pRL-SV40 encoding Renilla luciferase (rLuc) (Promega). Tandem repeat of ARE was inserted into the pGL6 luciferase reporter vector (ARE-pGL6-luc) (Beyotime). MIHA cells were co-transfected the ARE-pGL6-luc and pRL-SV40 encoding Renilla luciferase (rLuc) (Promega) before receiving FFA treatment. Control cells were co-transfected with empty pGL3-basic luciferase reporter vector and pRL-SV40. Dual-Luciferase assay was performed with Dual-Luciferase assay reagents (Promega). The luciferase readings for each sample were normalized against the rLuc levels. All samples were analyzed in triplicate in each individual experiment.

### Measurement of reactive oxygen species

The SchB-treated or control cells in the black 96-well plates were loaded with fluorescent dyes, 6-Carboxy-2′,7′-dichlorofluorescein diacetate. Fluorescence was measured using a fluorescence microplate reader (EnVision Multilabel Reader, PerkinElmer, Waltham, MA) at the indicated time point at 37°C. All samples were analyzed in triplicate in each individual experiment.

### Statistical analysis

The results are expressed as means ± standard error (SE). Statistically significant differences between two groups were assessed by Student's *t*-test.

## Author Contributions

L.A. and Y.Z.L. supervised the study. L.A., Y.Z.L., T.T., F.W.F. and K.H.Y. participated in study design. N.X. performed the IPA. D.W. performed the LCMS statistical analysis. K.H.Y., C.X., S.T., C.C.L., L.K.C., F.X. and T.A.K. performed the experiments and statistical analysis. P.S.Y. provided critical comments. K.H.Y. wrote the manuscript. All authors reviewed and approved the final manuscript.

## Supplementary Material

Supplementary Information Supplementary Information

## Figures and Tables

**Table 1:** SchB treatments affect plasma and hepatic TG levels in fasting and long-term HFD-fed mice

Mouse model (C57BL/6)	SchB	Treatment (Day)	Hepatic TG	Plasma TG
**Non-fasting, fed with regular diet**	0.8 g/kg	1	NS	NS
	50 mg/kg	1	NS	NS
**Overnight fasting after regular diet-fed**	0.8 g/kg	1	NS	Increased
	50 mg/kg	1	NS	NS
**High fat diet-fed**	50 mg/kg	20	Reduced	NS

NS: no significant effect compared with corresponding vehicle control mice; Increased: TG level was increased compared with corresponding vehicle control mice; Reduced: TG level was reduced compared with corresponding vehicle control mice

**Table 2:** The fatty acids and lipids metabolism canonical pathways of livers in non-fasting, fasting and HFD-fed mice

No.	Metabolism canonical pathways	Score of pathways (-log(*p*-value))		
		nf-veh vs. nf-schB	f-veh vs. f-schB	HFD-veh vs. HFD-schB
**1**	Phosphatidylethanolamine Biosynthesis III	5.11	4.95	5.11
**2**	Anandamide Degradation	2.44	2.36	2.44
**3**	Cardiolipin Biosynthesis II	2.09	2.01	2.09
**4**	Sphingomyelin Metabolism	1.90	1.82	1.90
**5**	Prostanoid Biosynthesis	1.87	6.30	1.87
**6**	Phosphatidylcholine Biosynthesis I	1.81	1.73	1.81
**7**	Phosphatidylethanolamine Biosynthesis II	1.79	1.71	1.79
**8**	Choline Biosynthesis III	1.70	3.59	3.75
**9**	Leukotriene Biosynthesis	1.70	5.78	1.70
**10**	γ-linolenate Biosynthesis II (Animals)	1.66	1.59	1.66
**11**	Triacylglycerol Degradation	1.60	1.52	1.60
**12**	Phosphatidylglycerol Biosynthesis II (Non-plastidic)	1.54	3.26	3.42
**13**	D-myo-inositol (1,4,5)-Trisphosphate Biosynthesis	1.49	1.41	1.49
**14**	Triacylglycerol Biosynthesis	1.42	3.02	3.18
**15**	Stearate Biosynthesis I(Animals)	1.37	2.91	3.07
**16**	CDP-diacylglycerol Biosynthesis I	0.0	1.55	1.63
**17**	Palmitate Biosynthesis I(Animals)	0.0	1.85	1.93
**18**	Phosphatidylethanolamine Biosynthesis III	5.11	4.95	5.11
**19**	Choline Biosynthesis III	3.81	3.75	1.92
**20**	Phosphatidylglycerol Biosynthesis II (Non-plastidic)	3.48	3.42	1.76
**21**	Triacylglycerol Biosynthesis	3.25	3.18	1.64
**22**	Cardiolipin Biosynthesis II	2.12	2.09	2.31
**23**	Sphingomyelin Metabolism	1.93	1.90	2.12
**24**	Phosphatidylcholine Biosynthesis I	1.84	1.81	2.03
**25**	Phosphatidylethanolamine Biosynthesis II	1.82	1.79	2.01
**26**	CDP-diacylglycerol biosynthesis I	1.66	1.63	0.0
**27**	Bile Acid Biosynthesis, Neutral Pathway	0.0	1.37	0.0
**28**	Superpathway of cholesterol biosynthesis	0.0	1.14	0.0
**29**	Cholesterol Biosynthesis I	0.0	1.46	0.0
**30**	Cholesterol Biosynthesis II (via 24,25-dihydrolanosterol)	0.0	1.46	0.0
**31**	Anandamide Degradation	0.0	2.44	0.0
**32**	Cholesterol Biosynthesis III(via Desmosterol)	0.0	1.46	0.0
**33**	Palmitate Biosynthesis I(Animals)	0.0	1.93	0.0
**34**	Triacylglycerol Degradation	0.0	1.60	1.82

*nf-veh*: non-fasting regular diet-fed vehicle control group; *nf-schB*: non-fasting regular-diet SchB-treated group; *f-veh*: fasting after regular diet-fed vehicle control group; *f-schB*: fasting after regular diet-fed SchB-treated group; *HFD-veh*: HFD-fed vehicle control group; *HFD-schB*: HFD-fed SchB-treated group

**Table 3:** Hepatic palmitic acid levels in non-fasting, fasting and HFD-fed mice

	Hepatic Palmitic Acid (μg/g)
**nf-veh**	13.3386 ± 0.098
**nf-SchB**	12.9917 ± 0.118

	Hepatic Palmitic Acid (μg/g)
**f-veh**	15.943 ± 0.640
**f-SchB**	14.847 ± 0.527

	Hepatic Palmitic Acid (μg/g)
**HFD-veh**	19.983 ± 0.379
**HFD-SchB**	17.534 ± 0.222*

*nf-veh*: non-fasting regular diet-fed vehicle control group; *nf-schB*: non-fasting regular-diet SchB-treated group; *f-veh*: fasting after regular diet-fed vehicle control group; *f-schB*: fasting after regular diet-fed SchB-treated group; *HFD-veh*: HFD-fed vehicle control group; *HFD-schB*: HFD-fed SchB-treated group. Shown is the mean ± SE (n = 10 mice), *p < 0.05 compared to vehicle control.

**Table 4:** Plasma palmitic acid levels in non-fasting, fasting and HFD-fed mice

	Plasma Palmitic Acid (μg/mL)
**nf-veh**	2.191 ± 0.584
**nf-SchB**	2.655 ± 0.658

	Plasma Palmitic Acid (μg/mL)
**f-veh**	7.466 ± 0.375
**f-SchB**	10.400 ± 0.129*

	Plasma Palmitic Acid (μg/mL)
**HFD-veh**	7.884 ± 0.325
**HFD-SchB**	6.430 ± 0.857

*nf-veh*: non-fasting regular diet-fed vehicle control group; *nf-schB*: non-fasting regular-diet SchB-treated group; *f-veh*: fasting after regular diet-fed vehicle control group; *f-schB*: fasting after regular diet-fed SchB-treated group; *HFD-veh*: HFD-fed vehicle control group; *HFD-schB*: HFD-fed SchB-treated group. Shown is the mean ± SE (n = 10 mice), *p < 0.05 compared to vehicle control.
