# Supplementary material for: Lipidomic-based investigation into the regulatory effect of Schisandrin B on palmitic acid level in non-alcoholic steatotic livers
Source: Sci Rep. 2015 Mar 13;5:9114. doi: 10.1038/srep09114 (PMC4358044; doi:10.1038/srep09114)
Supplement: Supplementary Information [file srep09114-s1.pdf]

Lipidomic-based investigation into the regulatory effect of Schisandrin B on palmitic acid level in non-alcoholic steatotic livers

Hiu Yee Kwan[1,2], Xuyan Niu[3], Wenlin Dai[4], Tiejun Tong[4], Xiaojuan Chao[1,2], Tao Su[1,2], Chi Leung Chan[1], Kim Chung Lee[5,6], Xiuqiong Fu[1,2], Yi Hua[7], Yu Hua[1,2], Li Ting[1,2], Anfernee Kai Wing Tse[1,2], Wang Fun Fong[1,2], Si-Yuan Pan[8], Aiping Lu[1,2§], Zhi-Ling Yu[1,2§]

Supplementary Figure S1

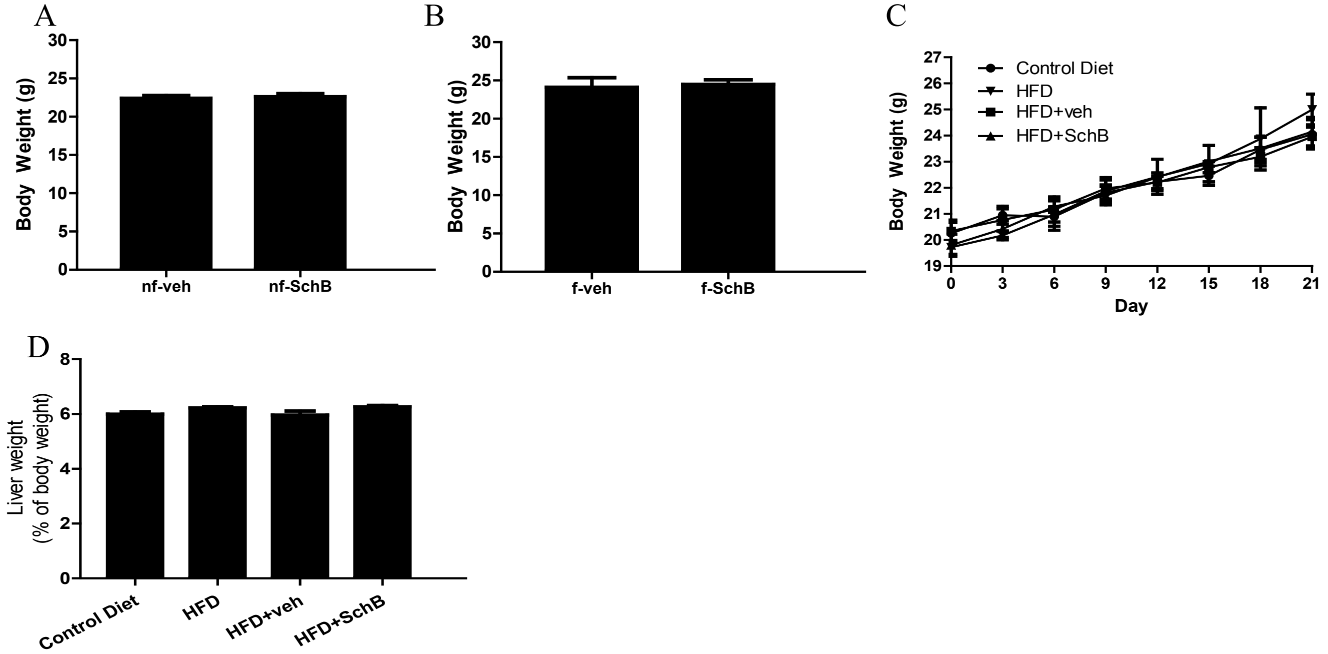

Supplementary Figure S1. Body weights of mice in **(A)** non-fasting group (nf), **(B)** fasting group (f) and **(C)** HFD-fed group (HFD). **(D)** Liver weights of HFD-fed mice. *nf-veh*: non-fasting vehicle control group; *nf-SchB*: non-fasting SchB-treated group; *f-veh*: fasting vehicle control group; *f-SchB*: fasting SchB-treated group; *HFD-veh*: HFD-fed vehicle control group; *HFD-SchB*: HFD-fed SchB-treated group. Shown is the mean ± SE (n=10 mice).

Supplementary Figure S2

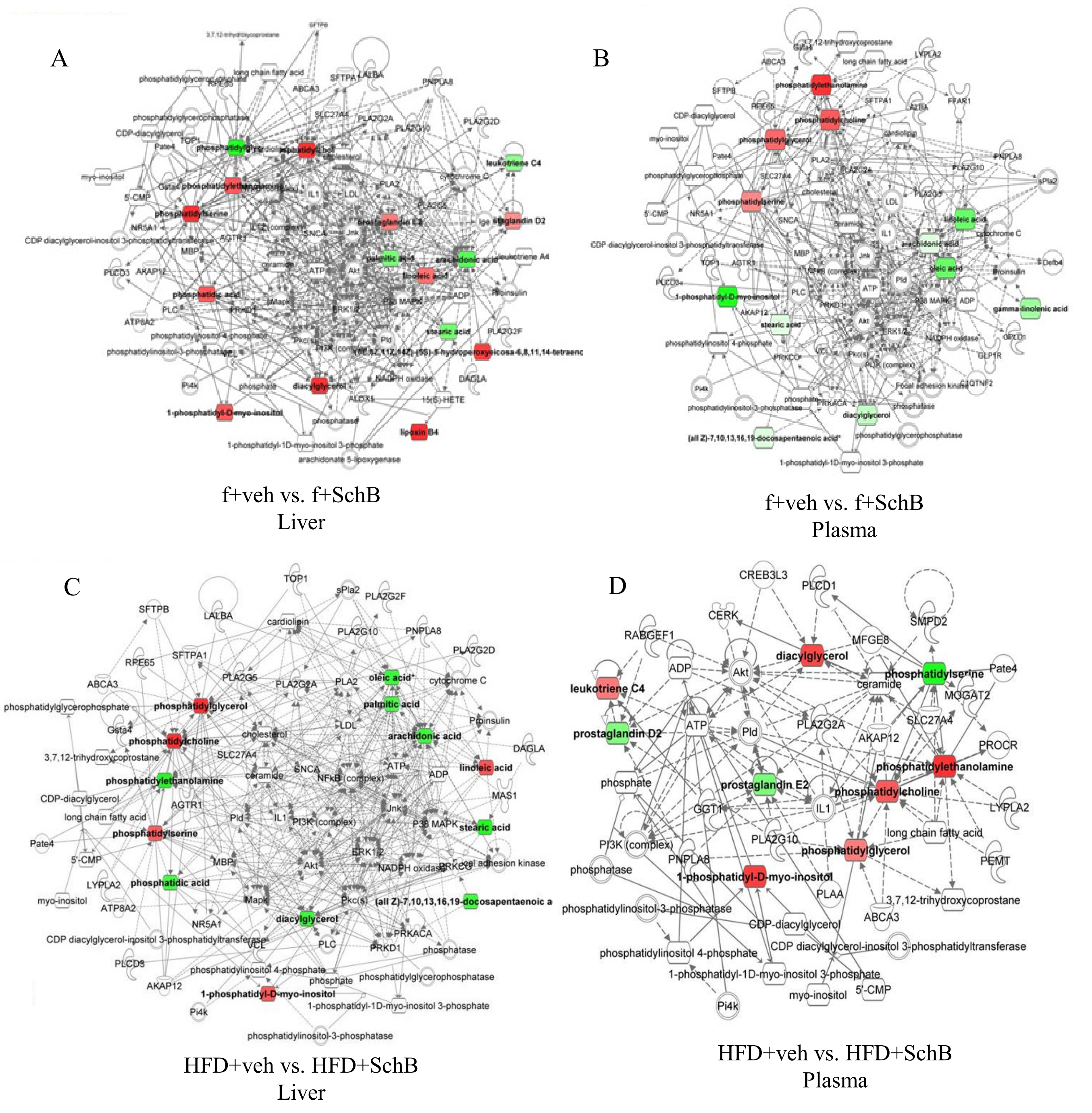

f+veh vs. f+SchB
Liver

f+veh vs. f+SchB
Plasma

HFD+veh vs. HFD+SchB
Liver

HFD+veh vs. HFD+SchB
Plasma

Supplementary Figure S2. Molecular network built based on the lipid species in **(A)** liver and **(B)** plasma samples in fasting mice; and **(C)** liver and **(D)** plasma samples in HFD-fed mice. Lipid entities are represented as nodes, and the biological relationship between two nodes is represented as a line. Colored symbols represents the lipid entities occurred in our analysis, transparent entries are the entities from Ingenuity Knowledge Database. Red symb ols represent up -regulated entities, green symbols represents down-regulated entities. Solid lines between the lipid entities indicate a direct physical relationship between the entities, dotted lines indicate indirect functional relationship. *f-veh*: fasti ng vehicle control group; *f-SchB*: fasting SchB-treated group; *HFD-veh*: HFD-fed vehicle control group; *HFD-SchB*: HFD-fed SchB-treated group

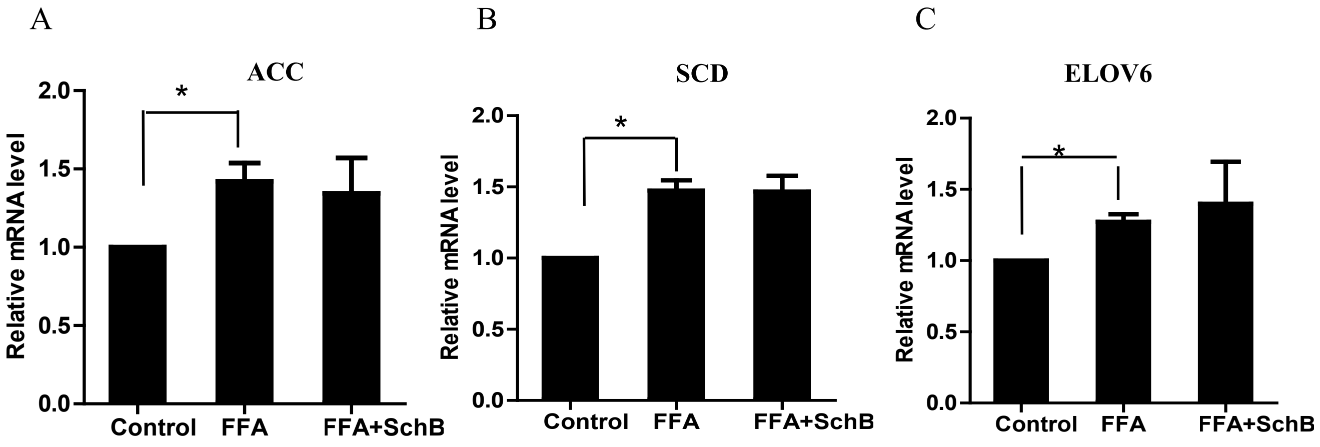

Supplementary Figure S3. The relative mRNA expressions of **(A)** acetyl CoA carboxylase (ACC), **(B)** stearoyl-CoA desaturase-1(SCD) and **(C)** elongation of long chain fatty acids family member 6 (ELOV6) in FFA-treated MIHA cells. Shown is the mean ± SE (n=3 independent experiments), **p*<0.05.

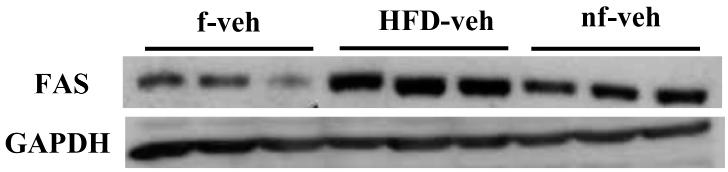

Supplementary Figure S4. Hepatic protein expressions of fatty acid synthase (FAS) in fasting, non-fasting and HFD-fed mice. *nf-veh*: non-fasting vehicle control group; *f-veh*: fasting vehicle control group; *HFD-veh*: HFD-fed vehicle control group

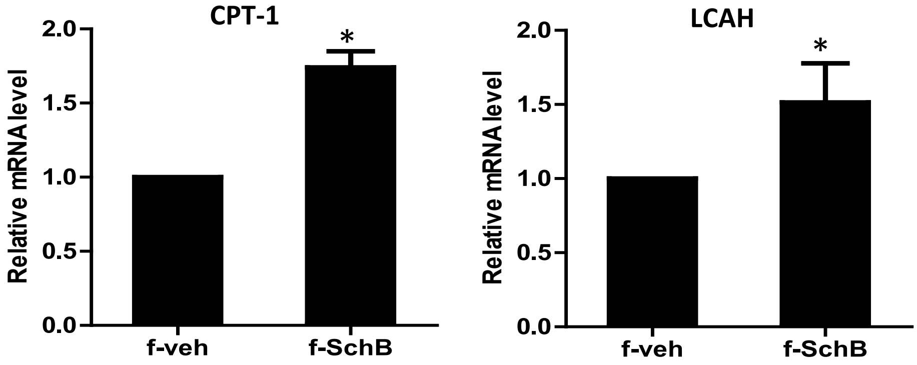

Supplementary Figure S5. Relative mRNA expression of carnitine palmitoyltransferase-1 (CPT-1) and very-long-chain acyl CoA dehydrogenase (LCAH) in fasting mice. *f-veh*: fasting vehicle control group; *f-SchB*: SchB-treated fasting group. Shown is the mean ± SE (n=4 mice), **p*<0.05.

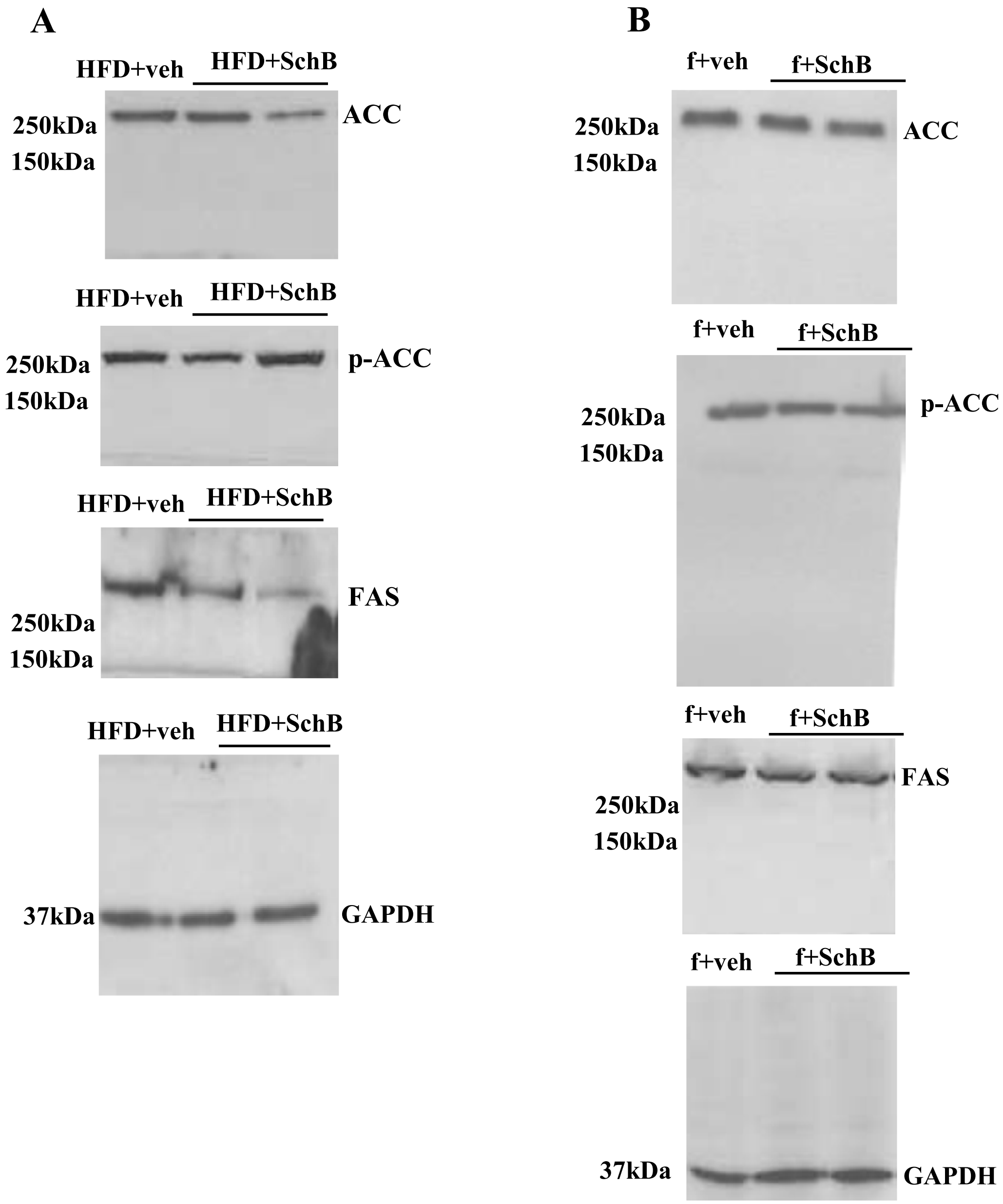

Supplementary S6 SchB treatment affects lipogenic gene expressions in long-term HFD-fed mice
Western blotting showing the expressions of acetyl CoA carboxylase (ACC), phospho-ACC (ser-563) and fatty acid synthase (FAS) in (A) HFD-fed mouse livers, (B) fasting mouse livers and (C) non-fasting mouse livers.. nf-veh: non-fasting vehicle control group; nf-SchB: non-fasting SchB-treated group; f-veh: fasting vehicle control group; f-SchB: fasting SchB-treated group; HFD-veh: HFD-fed vehicle control group; HFD-SchB: HFD-fed SchB-treated group. (G) expressions of ACC, phospho-ACC (ser-563) and FAS in FFA-treated MIHA cells. FFA: FFA-treated vehicle control MIHA cells; FFA+SchB: FFA-treated SchB-treated MIHA cells.

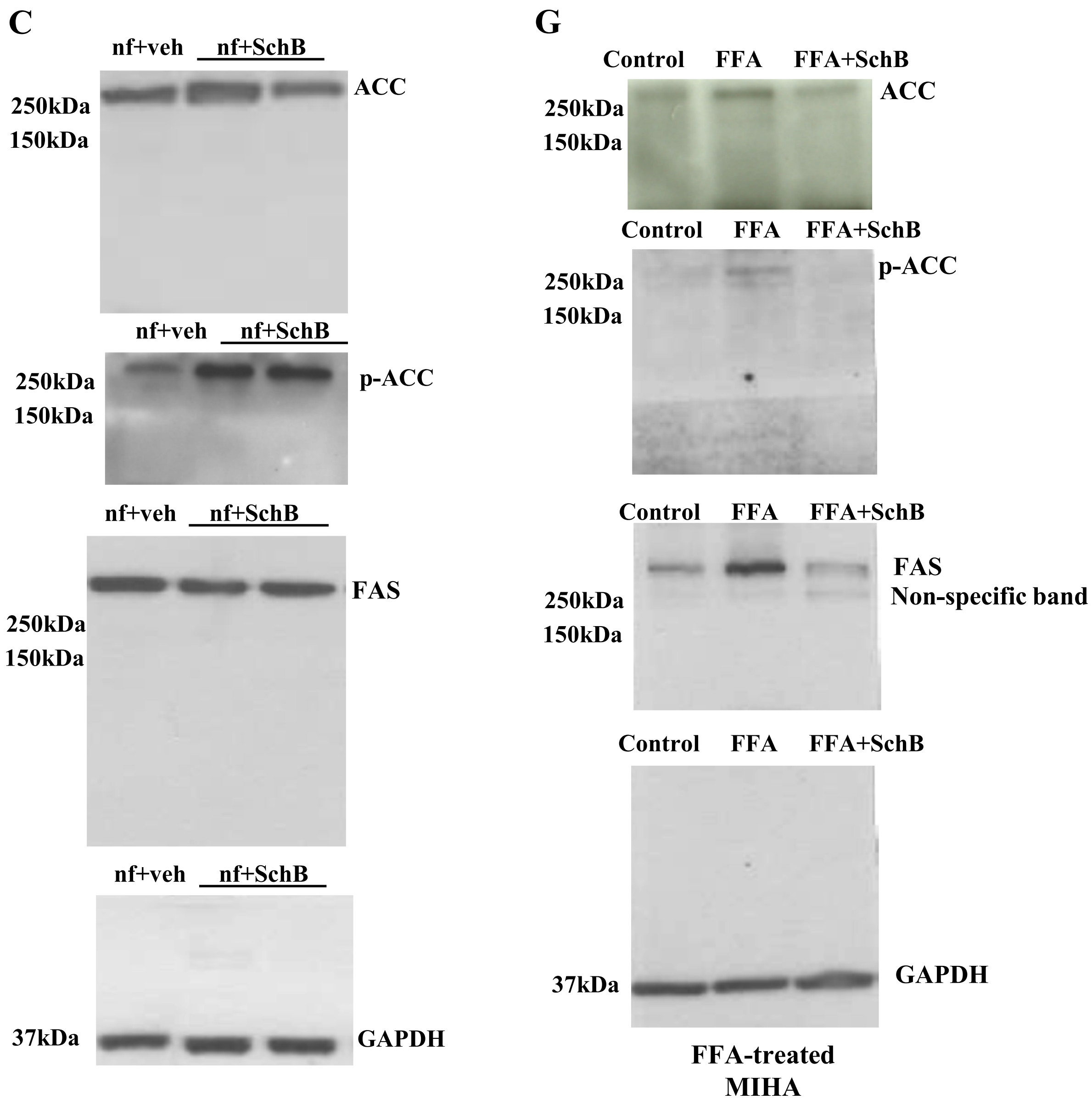

Supplementary S6 SchB treatment affects lipogenic gene expressions in long-term HFD-fed mice
Western blotting showing the expressions of acetyl CoA carboxylase (ACC), phospho-ACC (ser-563) and fatty acid synthase (FAS) in (A) HFD-fed mouse livers, (B) fasting mouse livers and (C) non-fasting mouse livers.. nf-veh: non-fasting vehicle control group; nf-SchB: non-fasting SchB-treated group; f-veh: fasting vehicle control group; f-SchB: fasting SchB-treated group; HFD-veh: HFD-fed vehicle control group; HFD-SchB: HFD-fed SchB-treated group. (G) expressions of ACC, phospho-ACC (ser-563) and FAS in FFA-treated MIHA cells. FFA: FFA-treated vehicle control MIHA cells; FFA+SchB: FFA-treated SchB-treated MIHA cells.

Supplementary Figure S7

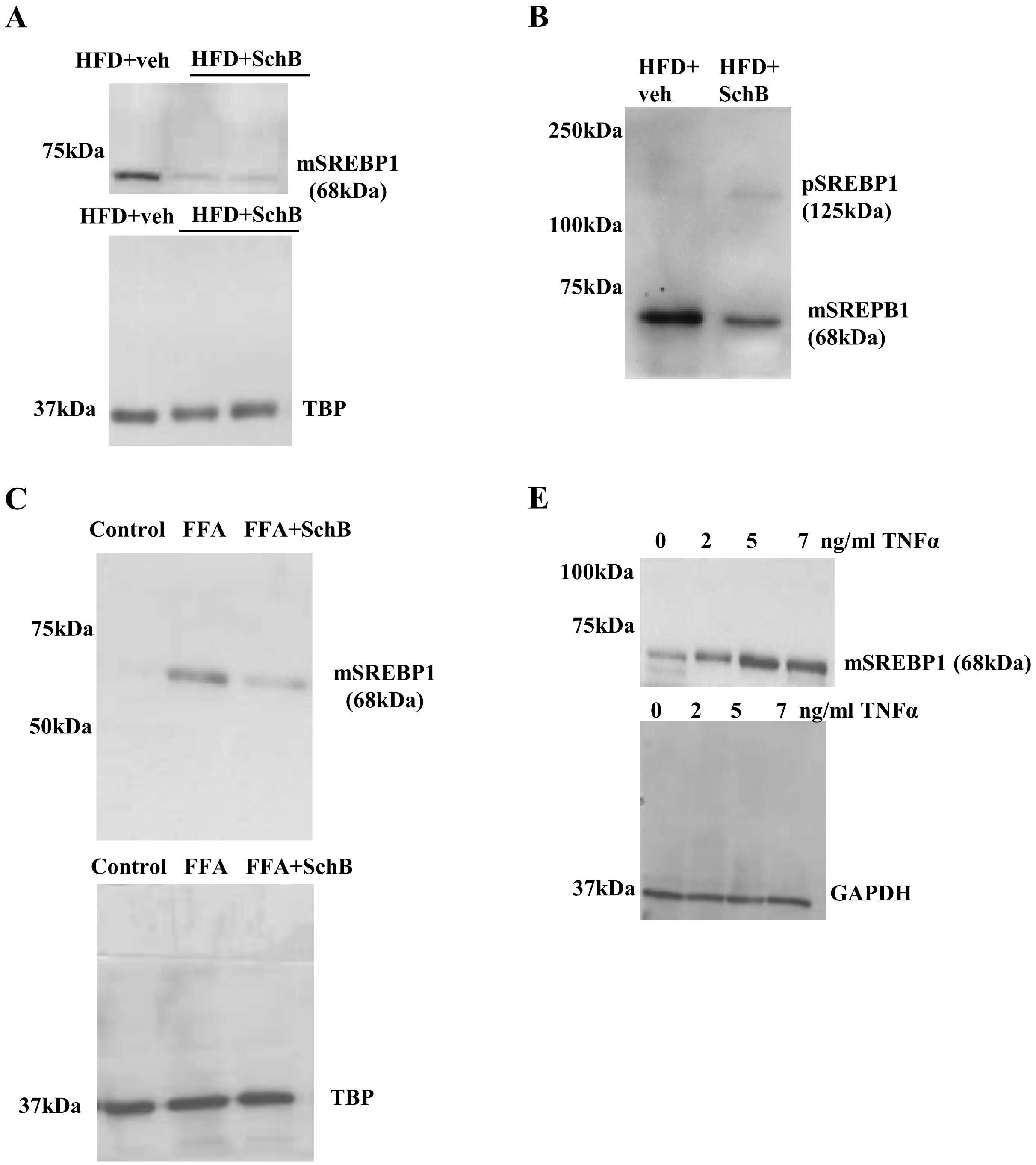

Supplementary S7 SchB treatment affects SREBP-1 expressions in long-term HFD-fed mice
Western blotting showing (A) expression of mature sterol regulatory element binding protein-1 (mSREBP-1), (B) ratio of precursor to mature SREBP-1 and (I) tumor necrosis factor (TNF-α) in HFD-fed mouse livers. (C) Protein expression of mature SREBP-1 (mSREBP-1) in FFA-treated MIHA cells. FFA: FFA-treated vehicle control MIHA cells; FFA+SchB: FFA-treated SchB-treated MIHA cells. Protein expressions of (E, G) mSREBP-1 and (F) FAS upon TNF-α challenges, and (H) protein expressions of TNF-α in FFA-treated MIHA cells. FFA: FFA-treated vehicle control MIHA cells; FFA+SchB: FFA-treated SchB-treated MIHA cells

(Cont'd) Supplementary Figure S7

F

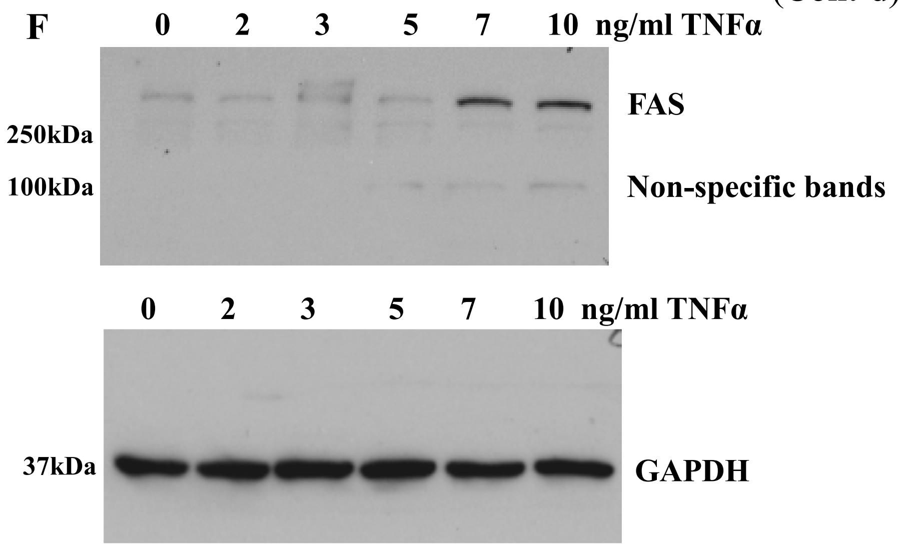

G

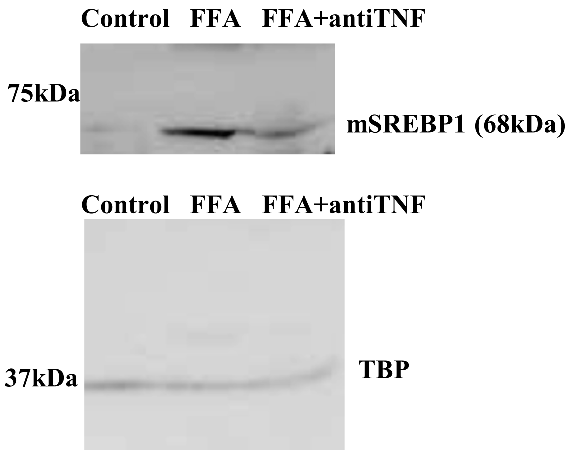

H

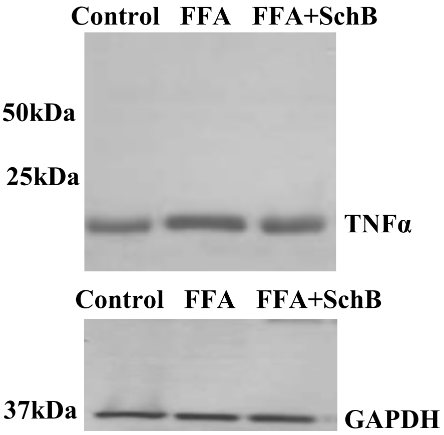

I

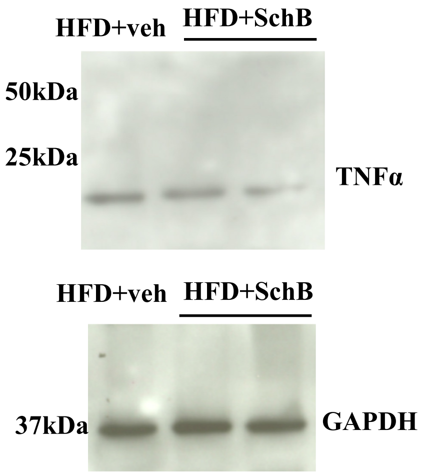

Supplementary S7 SchB treatment affects SREBP-1 expressions in long-term HFD-fed mice

Western blotting showing (A) expression of mature sterol regulatory element binding protein-1 (mSREBP-1), (B) ratio of precursor to mature SREBP-1 and (I) tumor necrosis factor (TNF-α) in HFD-fed mouse livers. (C) Protein expression of mature SREBP-1 (mSREBP-1) in FFA-treated MIHA cells. FFA: FFA-treated vehicle control MIHA cells; FFA+SchB: FFA-treated SchB-treated MIHA cells. Protein expressions of (E, G) mSREBP-1 and (F) FAS upon TNF-α challenges, and (H) protein expressions of TNF-α in FFA-treated MIHA cells. FFA: FFA-treated vehicle control MIHA cells; FFA+SchB: FFA-treated SchB-treated MIHA cells

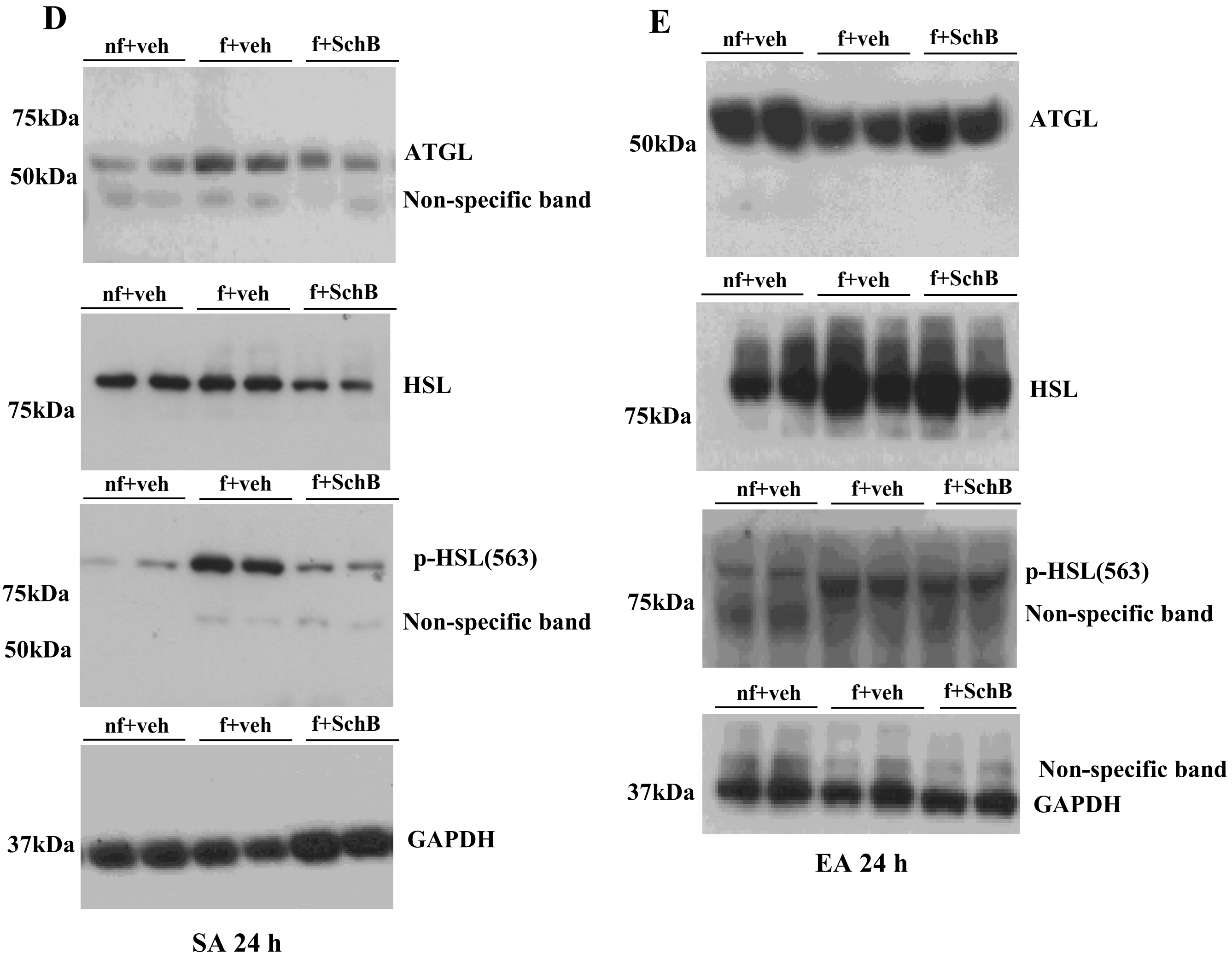

Supplementary S8 SchB treatment induces transient lipolysis in fasting mice

Western blotting showing the expressions of adipose triglyceride lipase (ATGL), hormone sensitive lipase (HSL), phospho-HSL (Ser-563) in adipocytes isolated from the bilateral superficial subcutaneous adipose tissue (SA), prominent bilateral intra-abdominal visceral depots attached to the epididymides (EA) and the perirenal fat (RA) (D-E) 24 h after fasting, (F) 2 h after fasting, (G) 6 h after fasting and (H) 12 h after fasting. nf-veh: non-fasting vehicle control group; nf-SchB: non-fasting SchB-treated group; f-veh: fasting vehicle control group; f-SchB: fasting SchB-treated group; HFD-veh: HFD-fed vehicle control group; HFD-SchB: HFD-fed SchB-treated group.

F

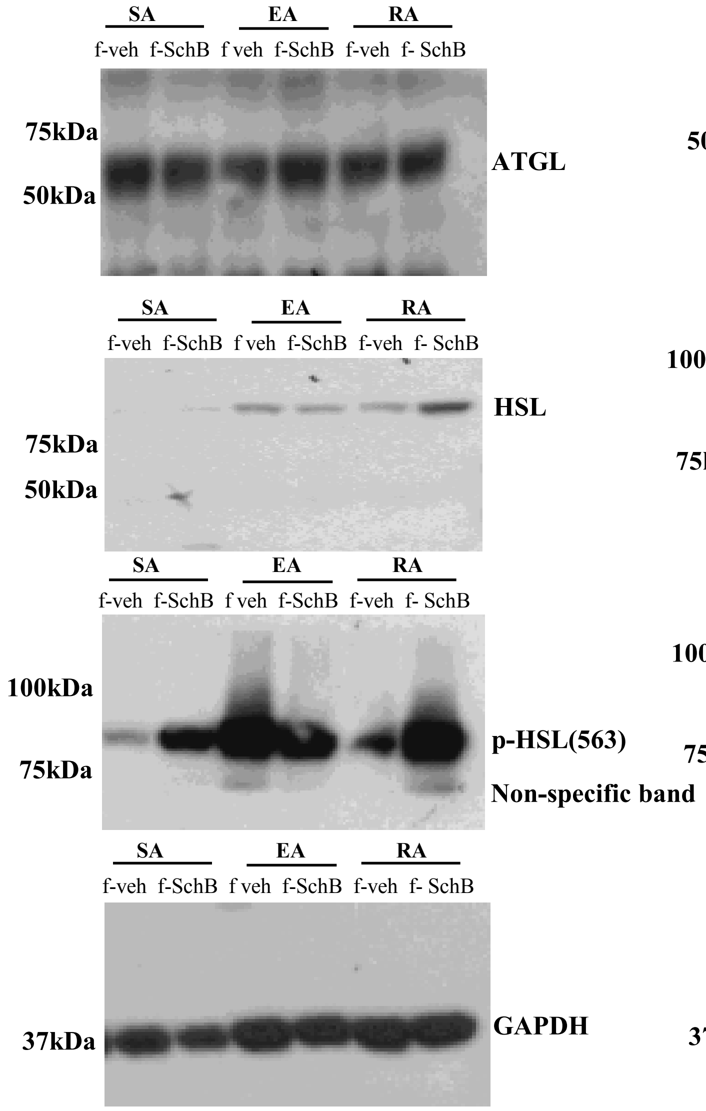

Fasting 2 h

G

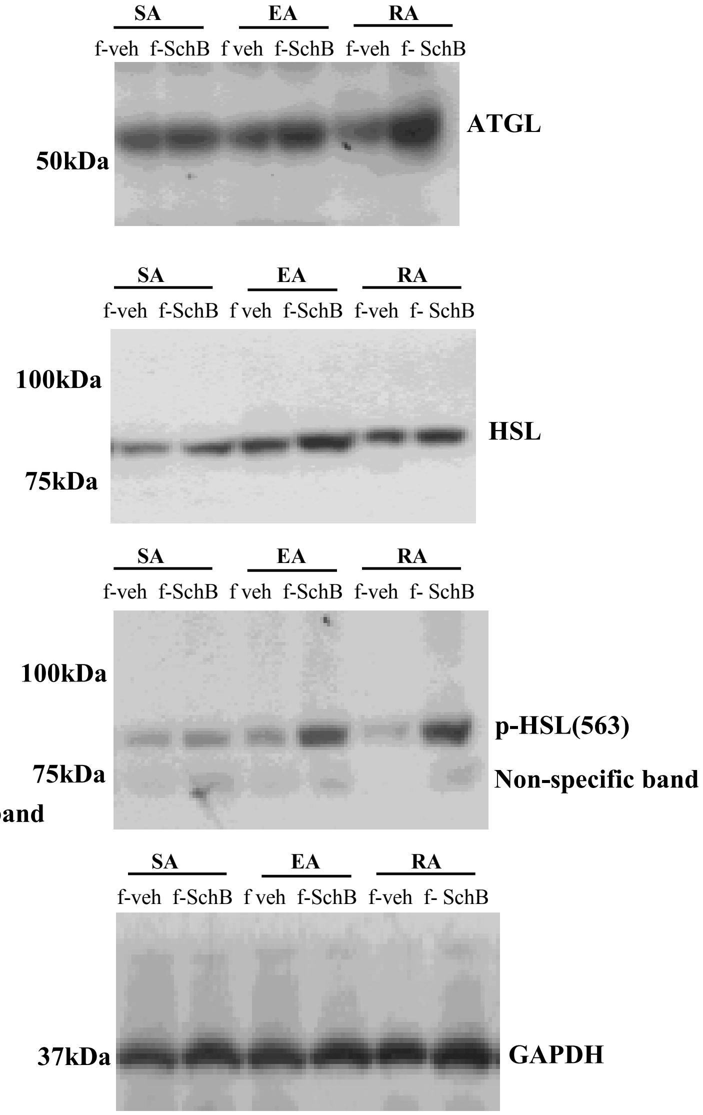

Fasting 6 h

Supplementary S8 SchB treatment induces transient lipolysis in fasting mice

Western blotting showing the expressions of adipose triglyceride lipase (ATGL), hormone sensitive lipase (HSL), phospho-HSL (Ser-563) in adipocytes isolated from the bilateral superficial subcutaneous adipose tissue (SA), prominent bilateral intra-abdominal visceral depots attached to the epididymides (EA) and the perirenal fat (RA) (D-E) 24 h after fasting, (F) 2 h after fasting, (G) 6 h after fasting and (H) 12 h after fasting. nf-veh: non-fasting vehicle control group; nf-SchB: non-fasting SchB-treated group; f-veh: fasting vehicle control group; f-SchB: fasting SchB-treated group; HFD-veh: HFD-fed vehicle control group; HFD-SchB: HFD-fed SchB-treated group.

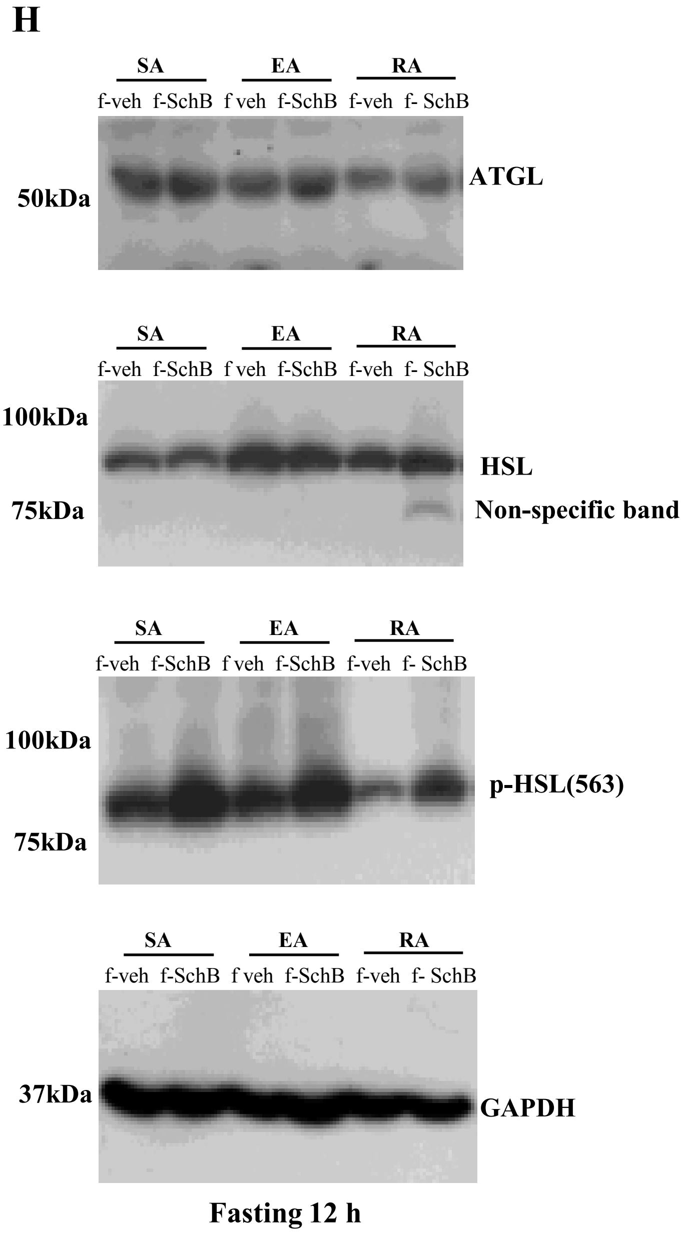

Supplementary S8 SchB treatment induces transient lipolysis in fasting mice

Western blotting showing the expressions of adipose triglyceride lipase (ATGL), hormone sensitive lipase (HSL), phospho-HSL (Ser-563) in adipocytes isolated from the bilateral superficial subcutaneous adipose tissue (SA), prominent bilateral intra-abdominal visceral depots attached to the epididymides (EA) and the perirenal fat (RA) (D-E) 24 h after fasting, (F) 2 h after fasting, (G) 6 h after fasting and (H) 12 h after fasting. nf-veh: non-fasting vehicle control group; nf-SchB: non-fasting SchB-treated group; f-veh: fasting vehicle control group; f-SchB: fasting SchB-treated group; HFD-veh: HFD-fed vehicle control group; HFD-SchB: HFD-fed SchB-treated group.

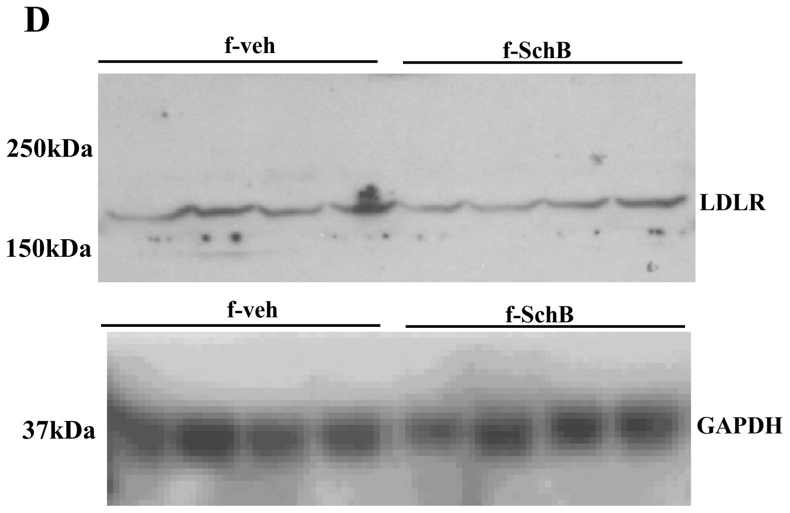

Liver

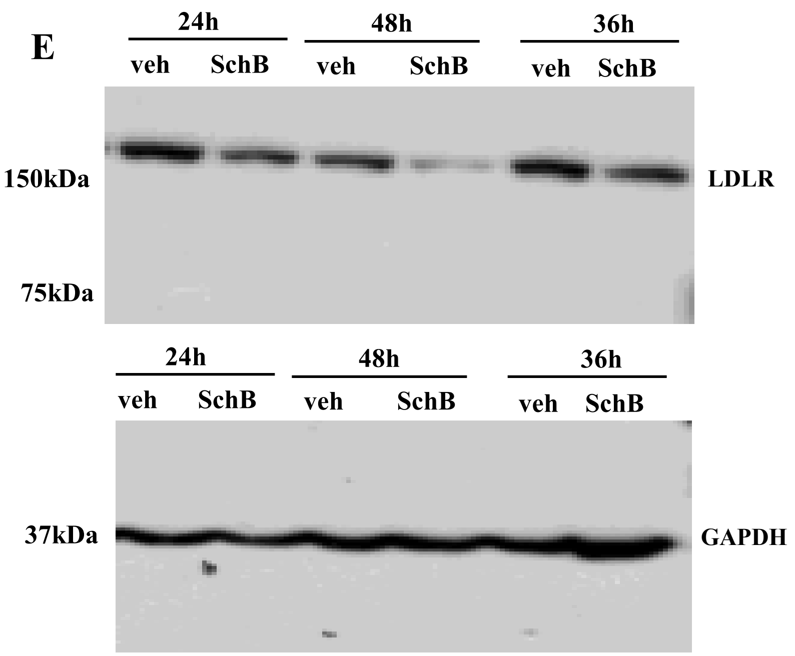

FFA-treated MIHA

Supplementary S9 SchB treatment affects the plasma cholesterol levels in fasting mice
Expressions of LDL receptor in (D) and in (E) FFA-treated MIHA cells. f-veh: fasting vehicle control group; f-SchB: fasting SchB-treated group.

Supplementary Table S1 The chromatographic and mass spectrometric parameters for the LC/MS

Chromatographic parameters	
Column	Waters Acquity UPLC BEH C18, 2.1x100mm, 1.7μM
Column temperature	45°C
Autosampler temperature	10°C
Mobile phase	A= Water with 10mM Ammonium formate pH4 adjusted by formic acid<br>B= Acetonitrile
Gradient	0% B (0-0.25min)<br>5%B (0.25-1min)<br>20%B (1-10min)<br>60%B (10-22min)<br>99%B (22-26min)
Flow rate	0.45 mL/min
Injection volume	12μl
**MS parameters**	
Polarity	Negative
Capillary voltage	4.0kV
Sheath gas flow rate	10 L/min
Sheath gas temperature	350°C
Drying gas flow rate (nitrogen)	7 L/min
Drying gas temperature	300°C
Nebuilizer gas (nitrogen)	45 psi
Fragmentor voltage	140V
Nozzle voltage	0V
Scanning range	50-1700 m/z

An Agilent 6540 UHD Accurate-Mass Q-TOF LC/MS mass spectrometer (Agilent Technologies) was connected to an Agilent 1290 Infinity UHPLC via an ESI ion source for the lipids analysis.

Supplementary Table S2A. The metabolism canonical pathways and identified metabolites in livers of fasting and HFD-fed mouse models

	ID	Identified lipid species	Formula	Regulation directions		Metabolism canonical pathways
				f-schB /f-ctl	HFD-SchB/HFD-veh	
1	C02737	Phosphatidylethanolserine	C13H24NO10P	↑	↑	Phosphatidylethanolamine Biosynthesis Ⅲ
2	C00344	Phosphatidylglycerol	C8H13O10PR2	↓	↑	Cardiolipin Biosynthesis Ⅱ; Phosphatidylglycerol Biosynthesis Ⅱ
3	C00157	Phosphatidylcholine	C10H18NO8PR2	↑	↑	Sphingomyelin Metabolism; Choline Biosynthesis II; Phosphatyidylcholine Biosynthesis I; Triacylglycerol Biosynthesis
4	C00219	Arachidonic acid	C20H32O2	↓	↓	Prostanoid Biosynthesis; Leukotriene Biosynthesis; Anandamide Degradation
5	C00712	Oleic acid	C18H34O2	NS	↓	Oleate Biosynthesis
6	C00350	Phosphatidylethanolamine	C7H12NO8PR2	↑	↓	Phosphatidylethanolamine Biosynthesis
7	C05356	5(S)-HPETE	C20H32O4	↑	NS	Leukotriene Biosynthesis
8	C02166	Leukoteiene C4	C30H47N3O9S	↓	NS	Leukotriene Biosynthesis
9	C01595	Linoleic acid	C18H32O2	↑	↑	γ-linolenate Biosynthesis Ⅱ
10	C00165	Diacylglycerol	C5H6O5R2	↑	↓	Triacylglycerol Degradation
11	C00416	Phosphatidic acid	C5H7O8PR2	↑	↓	Phosphatidylglycerol Biosynthesis II; Choline Biosynthesis Ⅲ;Triacylglycerol Biosynthesis; CDP-diacylglycerol Biosynthesis I
12	C01194	1-phosphatidyl-D-myoinositol	C11H17O13PR2	↑	↑	D-myo-inositol (1,4,5)-Trisphosphate Biosynthesis
13	C01530	Stearic acid	C18H36O2	↓	↓	Stearate BiosynthesisI
14	C00249	Palmitic acid	C16H32O2	↓	↓	Palmitate Biosynthesis

f-veh: fasting after regular diet-fed vehicle control group; f-schB: fasting after regular diet-fed SchB-treated group; HFD-veh: HFD-fed vehicle control group; HFD-schB: HFD-fed SchB-treated group

Supplementary Table S2B. The metabolism canonical pathways and identified metabolites in plasma of fasting and HFD-fed mouse models

	ID	Identified lipid species	Formula	Regulation directions f-schB /f-ctl	Regulation directions HFD-SchB/HFD-veh	Metabolism canonical pathways
1	C00350	Phosphatidylethanolamine	C7H12NO8PR2	↓	↑	Phosphatidyl-ethanolamine Biosynthesis
2	C02737	Phosphatidylserine	C8H12NO10PR2	↓	↓	Phosphatidyl-ethanolamine Biosynthesis
3	C00157	Phosphatidylcholine	C10H18NO8PR2	↑	↑	Choline Biosynthesis Ⅲ;Triacylglycerol Biosynthesis; Sphingomyelin Metabolism; Phosphatidylcholine Biosynthesis Ⅰ
4	C00416	Phosphatidic acid	C5H7O8PR2	↑	NS	Choline Biosynthesis Ⅲ; Phosphatidylglycerol; Triacylglycerol Biosynthesis; CDP-diacylglycerol biosynthesis Ⅰ
5	C00344	Phosphatidylglycerol	C8H13O10PR2	↑	↑	Phosphatidylglycerol Biosynthesis Ⅱ; Cardiolipin Biosynthesis Ⅱ
6	(C00187	Cholesterol	C27H46O	↑	NS	Bile Acid Biosynthesis, Neutral Pathway; Cholesterol Biosynthesis Ⅰ; Cholesterol Biosynthesis Ⅱ(via 24,25-dihydrolanosterol); Cholesterol Biosynthesis Ⅲ(via Desmosterol)
7	C00219	Arachidonic acid	C20H32O2	↓	NS	Anandamide Degradation
8	C00249	Palmitic acid	C16H32O2	↑	NS	Palmitate Biosynthesis
9	C01530	Stearic acid	C18H36O2	↑	NS	Stearate Biosynthesis
10	C00165	Diacylglycerol	C5H6O5R2	↑	↑	Triacylglycerol degradation

f-veh: fasting after regular diet-fed vehicle control group; f-schB: fasting after regular diet-fed SchB-treated group; HFD-veh: HFD-fed vehicle control group; HFD-schB: HFD-fed SchB-treated group
